# Transcriptomic analysis of the testicular fusion in *Spodoptera litura*

**DOI:** 10.1186/s12864-020-6494-3

**Published:** 2020-02-19

**Authors:** Yaqing Chen, Jun Ou, Yucheng Liu, Qiong Wu, Liang Wen, Sichun Zheng, Sheng Li, Qili Feng, Lin Liu

**Affiliations:** 1 Guangdong Provincial Key Laboratory of Insect Developmental Biology and Applied Technology, Institute of Insect Science and Technology, School of Life Sciences, South China Normal University, Guangzhou, 510631 China; 2 Guangzhou Key Laboratory of Insect Development Regulation and Applied Research, Institute of Insect Science and Technology, School of Life Sciences, South China Normal University, Guangzhou, 510631 China

**Keywords:** Transcriptomic analysis, Testicular fusion, *Spodoptera litura*, Extracellular matrix (ECM), Matrix metalloproteinase (MMP)

## Abstract

**Background:**

Lepidoptera is one group of the largest plant-feeding insects and *Spodoptera litura* (Lepidoptera: Noctuidae) is one of the most serious agricultural pests in Asia countries. An interesting and unique phenomenon for gonad development of Lepidoptera is the testicular fusion. Two separated testes fused into a single one during the larva-to-pupa metamorphosis, which is believed to contribute to sperm production and the prevalence in field. To study the molecular mechanism of the testicular fusion, RNA sequencing (RNA-seq) experiments of the testes from 4-day-old sixth instar larvae (L6D4) (before fusion), 6-day-old sixth instar larvae (L6D6, prepupae) (on fusing) and 4-day-old pupae (P4D) (after fusion) of *S. litura* were performed.

**Results:**

RNA-seq data of the testes showed that totally 12,339 transcripts were expressed at L6D4, L6D6 and P4D stages. A large number of differentially expressed genes (DEGs) were up-regulated from L6D4 to L6D6, and then more genes were down-regulated from L6D6 to P4D. The DEGs mainly belongs to the genes related to the 20E signal transduction pathway, transcription factors, chitin metabolism related enzymes, the families of cytoskeleton proteins, extracellular matrix (ECM) components, ECM-related protein, its receptor integrins and ECM-remodeling enzymes. The expression levels of these genes that were up-regulated significantly during the testicular fusion were verified by qRT-PCR. The matrix metalloproteinases (MMPs) were found to be the main enzymes related to the ECM degradation and contribute to the testicular fusion. The testis was not able to fuse if MMPs inhibitor GM6001 was injected into the 5th abdomen region at L6D6 early stage.

**Conclusions:**

The transcriptome and DEGs analysis of the testes at L6D4, L6D6 and P4D stages provided genes expression information related to the testicular fusion in *S. litura*. These results indicated that cytoskeleton proteins, ECM-integrin interaction genes and ECM-related proteins were involved in cell migration, adhesion and fusion during the testicular fusion. The ECM degradation enzymes MMPs probably play a critical role in the fusion of testis.

## Background

Lepidoptera is one group of the largest plant-feeding insects. They are important in pollinating and preying and have substantial impacts on humans and many other species. Lepidopteran insects are also important for the studies of genetics, evolutionary biology, ecology, physiology and development [1]. *S. litura* is one of the most serious agricultural pests in the tropical and subtropical areas of Asia including India, China and Japan [1, 2]. The genome of *S. litura* has been sequenced and the genomic information provide a platform for further functional analysis [2].

Efficient reproduction depends on the production of health sperms and eggs during insect life cycle [3]. The male reproductive system of insects consists of the testes, vas deferens, seminal vesicles, accessory glands, single or double ejaculatory ducts, and aedeagus [4, 5]. An interesting and special phenomenon during the metamorphosis process is the testicular fusion, which occurs in most of the lepidopteran insects. In the larval period, a pair of kidneys-like testes are separated in the abdomen. During the prepupal or pupal period, the two separated testes fuse together to form a single one [6–8]. The testicular fusion has been reported in many lepidopteran insects, such as the Crambidae insects *Diatraea saccharalis* [7], the Lymantriidae insect *Agraulis vanillae* [8], the Nymphalidae insect *Dione juno* [8], the Noctuidae insects *Heliothis zea* [9], *Heliothis virescens* [10], and *S. litura* [11] and *Ostrinia nubilalis* [12], the Sphingidae insect *Manduca sexta* [13]. Most of these insects are important agricultural pests, causing extensive damage to cotton, soybean, tobacco, cruciferous vegetables [14, 15]. Comparatively, the Bombycidae insect *Bomby mori*, which is a domesticized lepidopteran insect, the testis does not fuse during the development [4]. Although there are many reports describing the process of testicular fusion, no study has reported on the molecular mechanism of testicular fusion in lepidopteran insects.

Fusion of cell or tissues is an indispensable process in diverse physiological events. Fusion events of tissue or organ are reported in the different developmental process in different organisms such as sperm-egg adhesion and fusion in mammals [16], myoblast fusion in flies and mice [17], epithelial fusion during neural tube morphogenesis [18] and medial cardiac cell fusion during the heart morphogenesis in *Drosophila* [19]. Fusion is essential for the fertilization, muscle development, neural tube formation and heart formation. Some reports have investigated the molecular mechanism of these fusion events. For example, the proteins ADAMs (a disintegrin and metalloprotease domain) including fertilin α, fertilin β and cyritestin, have been found to be important for sperm-egg binding and fusion by interacting with integrins on oocyte [20]. The major proteins involved in cell recognition and adhesion in mice are integrins, cadherins and focal adhesion proteins and the ECM are remodeled by MMPs during the myoblast fusion in the process of muscle regeneration [21–23]. During *Drosophila* heart development, cardioblasts (CBs) in the lateral mesoderm undergo specific medial adhesions with their contralateral partners, forming an apical lumen. MMPs promote the collective CB cell migration, ECM remodeling and lumen formation. Integrin and cadherin are also involved in cell adhesion and fusion [19]. These studies indicate that ECM remodeling is very important in the sperm-egg fusion, myoblast fusion and heart development of *Drosophila*, and the MMPs are the main enzymes in ECM remodeling [19, 23]. Although, the mechanisms of the fusion of different cells or tissues are reported in different species, the mechanism for the testis fusion in insects is still unknown.

In our previous study, a barrier sheet was embedded between the two separated testes so that the testicular fusion process was blocked. We found that the number of sperm bundless was decreased for those individuals that testes did not fuse at pupal stage. And the hatch rate of eggs was decreased if female mated with male whose testis did not fuse. It was speculated that testicular fusion benefit the development of sperms in lepidopteran insect [11]. It is interesting and compelling to figure out the mechanism of the testicular fusion, find key genes related to testicular fusion. The research will help us to understand the miracle of fusion process and develop the strategy for male sterility technology to control the pest in the future.

In this study, transcriptomic analysis of the testicular fusion in *S. litura* was conducted by RNA-seq technology [24] to examine the molecular process and potential genes that are involved in the fusion. The molecular mechanism of testicular fusion is discussed.

## Results

### RNA-sequencing and statistics of transcript expression

**Fig. 1:**
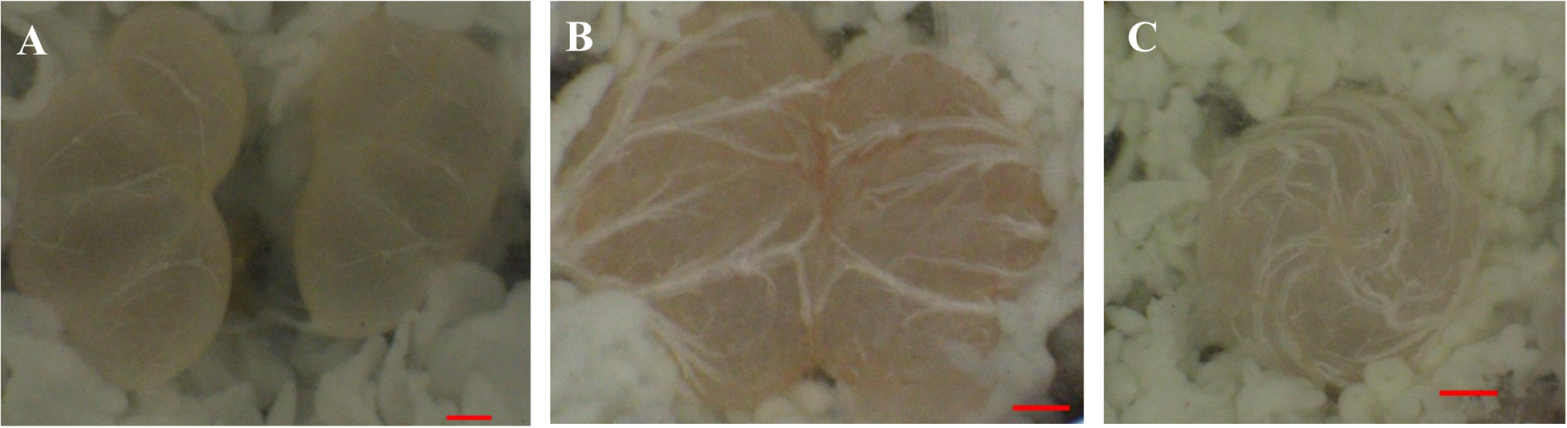
The testes of *S. litura* at different development stages. **a** Two separated testes at L6D4; **b** The fusing testes at L6D6 stage; **c** One testis after fusion at P3D. The red bars represent 100 μm

**Fig. 2:**
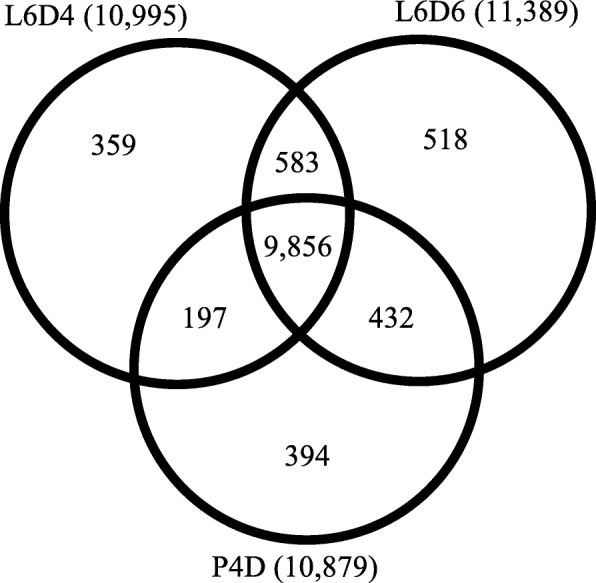
The read numbers of the expressed transcripts and distribution at L6D4, L6D6 and P4D testes

To explore the molecular mechanism of testicular fusion during the larva-to-pupa metamorphosis in *S. litura*, the samples of the testes were collected at L6D4, L6D6 and P4D (Fig. 1). The mRNA was extracted and sequenced by SE using Illumina Hiseq™ 2000 platform. In total, 4,780,380, 4,891,555 and 4,854,931 clean reads were obtained respectively for three groups of samples (Additional file 1). These clean reads were well mapped to reference sequences from *S. litura* genome database [2] using TOPHat software [25]. At L6D4, L6D6 and P4D stages 4,524,698, 4,602,274 and 4,594,593 unique reads were mapped and the mapping ratios were higher than 94% (Additional file 2). All the mapped clean reads were assembled by Cufflinks software [24] and the known genes from *S. litura* genome were obtained. Novel transcripts were detected using Cuffcompare. There were 12,339 transcripts expressing in all the three samples, while 9856 transcripts were co-expressed at the three stages, and 10,995, 11,389 and 10,879 transcripts were expressed at L6D4, L6D6 and P4D stages, respectively (Fig. 2).

**Fig. 3:**
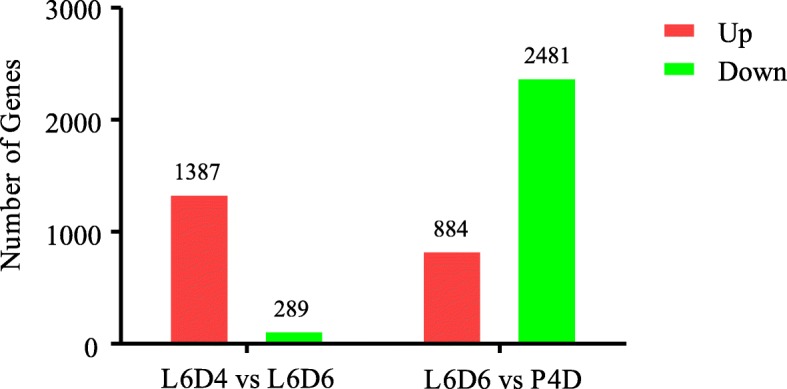
Comparison of the differentially expressed genes (DEGs) between L6D4 and L6D6, and between L6D6 and P4D. The red shows the up-regulated DEGs and the green show the down-regulated DEGs

The expression levels of transcripts were calculated by using the Fragments Per Kilobase of transcript per Million mapped reads (FPKMs) [24, 26]. According to FRKM, the differentially expressed genes (DEGs) were obtained comparing the samples between L6D4 and L6D6 (L6D4 vs L6D6), and between L6D6 and P4D (L6D6 vs P4D) by using the edgeR platform (http://www.bioconductor.org/packages/release/bioc/html/edgeR.html) [27]. In total, 1676 transcripts were identified as DEGs for L6D4 vs L6D6, of which 1387 were up-regulated and 289 were down-regulated. For L6D6 vs P4D, 3365 transcripts showed differential expression levels, of which 884 were up-regulated and 2481 were down-regulated (Fig. 3). The results indicated a large number of the transcripts were up-regulated during the testis fusion, but after the fusion, the major of DEGs were down-regulated. It is speculated that those transcripts that were expressed in such a trend may be involved in the testicular fusion.

### Expression of the 20-hydroxyecdysone (20E) and juvenile hormone (JH) signaling pathway related genes during the testicular fusion

**Fig. 4:**
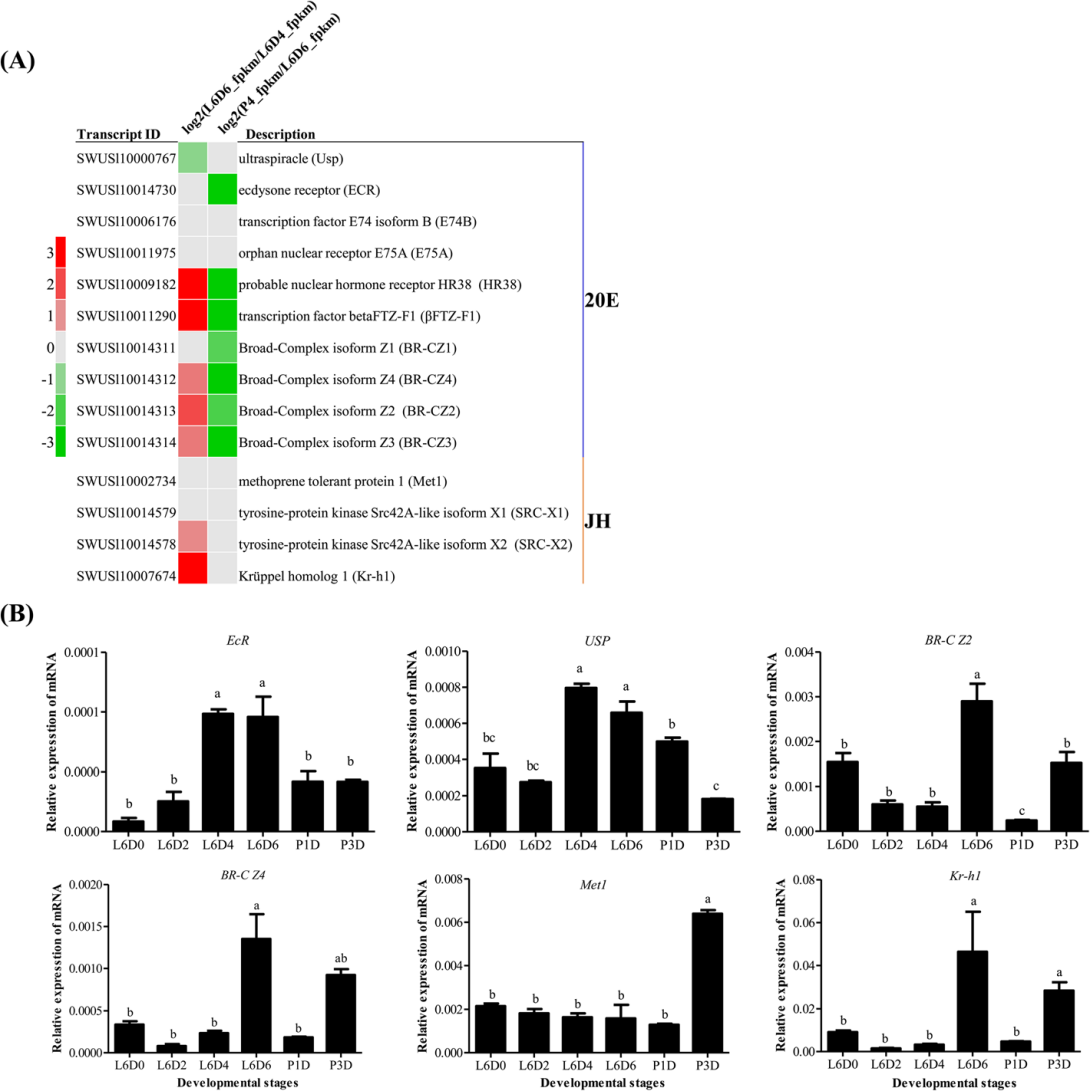
Expression of the 20E and JH signals-related genes. **a** Transcriptomic analysis of the expression of the 20E and JH signals-related genes; **b** qRT-PCR analysis of the expression of the 20E and JH signals-related genes in the testes at indicated six developmental stages in X-axis. L6D0, L6D2, L6D4 and L6D6 represent day 0, 2, 4 and 6 of the 6th instar larval stage; P1D and P3D represent day 1 and 3 after pupation. 20E signals genes includes *Ultraspiracle* (*USP*), *Ecdysone receptor* (*EcR*), *the nuclear hormone receptor 38* (*HR38*), the transcription factors *betaFTZ-F1*(*βFTZ-F1*)*, Broad Complex isform Z2/Z4* (*BRC-Z2/Z4*). JH signals genes includes *Methoprene-tolerant* (*Met1*) and *Krüppel homolog (Kr-h1*). The relative expression levels are normalized to the expression level of *Slgapdh*. One-way ANOVA analysis of variances was performed, followed by Turkey’s Multiple Comparison Test for significance analysis of the differences by GraphPad Prism 5 software. The values are mean ± SEM (*n* = 3). The significance is represented with different letters

The testicular fusion occurs during the larva-pupae metamorphosis, which is regulated by 20E and JH. In this study, the 20E signaling pathway related genes *Ultraspiracle* (*USP*), *Ecdysone receptor* (*EcR*), *the nuclear hormone receptor 38* (*HR38*), the transcription factors *betaFTZ-F1*(*βFTZ-F1*) and *Broad Complex isform Z2/Z3/Z4* (*BRC-Z2/Z3/Z4*) were highly expressed at L6D6 (Fig. 4a) and the genes *Krüppel homolog (Kr-h1*) and *Steroid receptor coactivator* (*SRC*) in JH signaling pathway were up-regulated at L6D6 (Fig. 4b). The results implied that the 20E signaling pathway may mainly regulate or initiate the testicular fusion, while JH signaling may also play roles in the process.

### Expression of transcription factors (TFs) during the testicular fusion

**Fig. 5:**
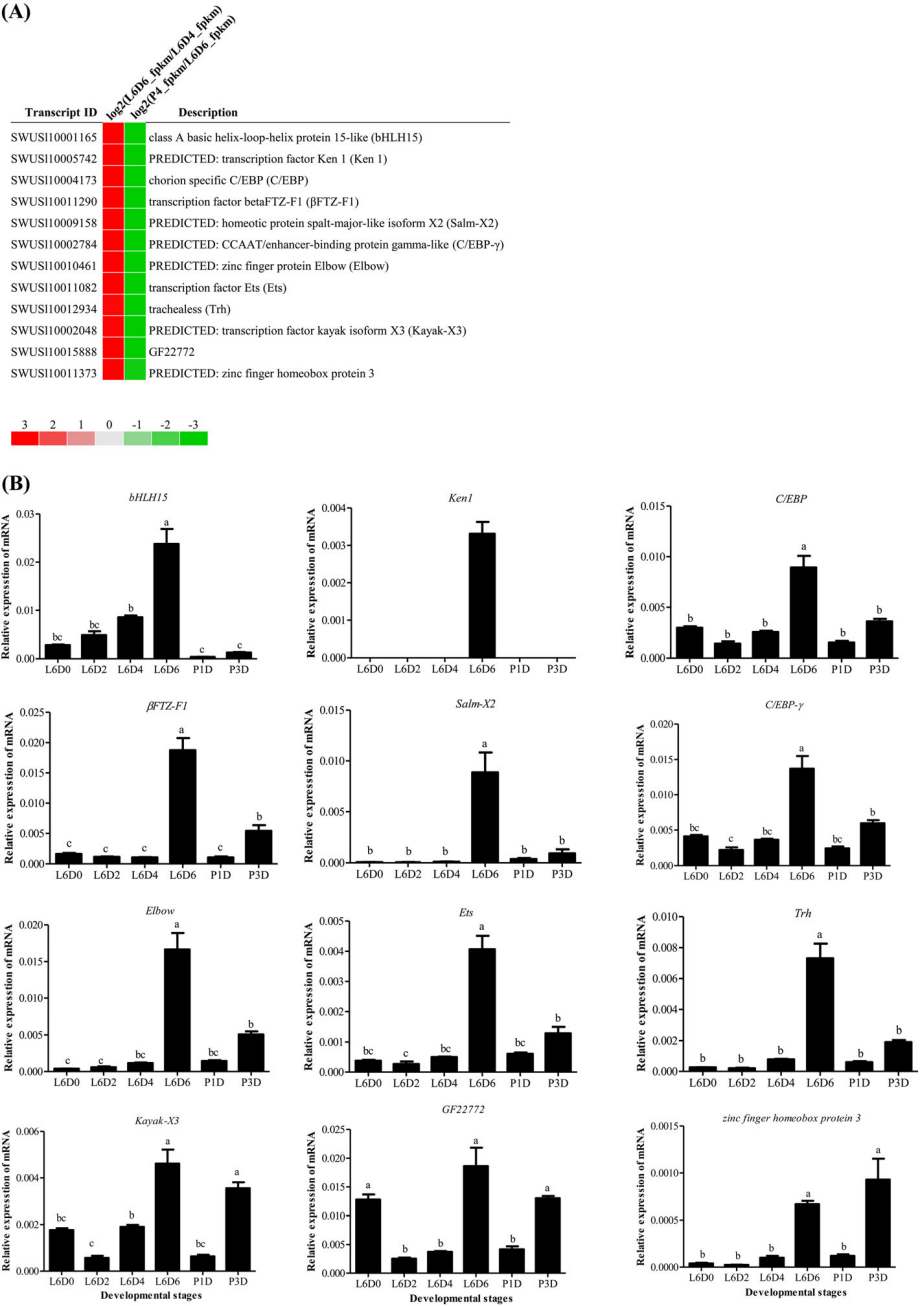
Expression of the selected 12 transcription factors. **a** Transcriptomic analysis of the expression of the 12 transcription factors; **b** qRT-PCR analysis of the expression of the 12 transcription factors at indicated six developmental stages of testes. The transcription factors includes *class A basic helix-loop-helix protein 15-like* (*bHLH15*), *Ken1*, *chorion specific C/EBP* (*C/EBP*), *transcription factor beta FTZ-F1* (*βFTZ-F1)*, *homeotic protein spalt-major-like isform X2* (*Salm-X2*), *CCAAT/enhancer-binding protein gamma-like* (*C/EBP-γ*), *zinc finger protein Elbow* (*Elbow*), *avian erythroblastosis virus E26 transformation-specific* (*Ets*), *trachealess* (*Trh*), *transcription factor kayak isform X3* (*Kayak-X3*), *GF22772* and *zinc finger homeobox protein 3* . The method and other information are the same as the Fig. 4. The significance is represented with different letters

Transcription factors regulate the growth, development and differentiation of organisms. By using Pfam (http://pfam.xfam.org/), a total of 187 transcripts with potential transcription factor activity were predicted in DEGs, 12 of which were specifically highly expressed at L6D6 (when the testicular fusion occurred) including the transcription factors *class A basic helix-loop-helix protein 15-like* (*bHLH15*), *Ken1*, *chorion specific C/EBP* (*C/EBP*), *βFTZ-F1*, *homeotic protein spalt-major-like isform X2* (*Salm-X2*), *CCAAT/enhancer-binding protein gamma-like* (*C/EBP-γ*), *zinc finger protein Elbow* (*Elbow*), *avian erythroblastosis virus E26 transformation-specific* (*Ets*), *trachealess* (*Trh*), *kayak isform X3* (*Kayak-X3*), *GF22772* and *zinc finger homeobox protein 3* (Fig. 5a). Their expression pattern was verified by qRT-PCR (Fig. 5b). It is hypothesized that highly expressed transcription factors at L6D6 may be involved in testicular fusion.

### Expression of the cytoskeleton and chitin metabolism related genes during the testicular fusion

**Fig. 6:**
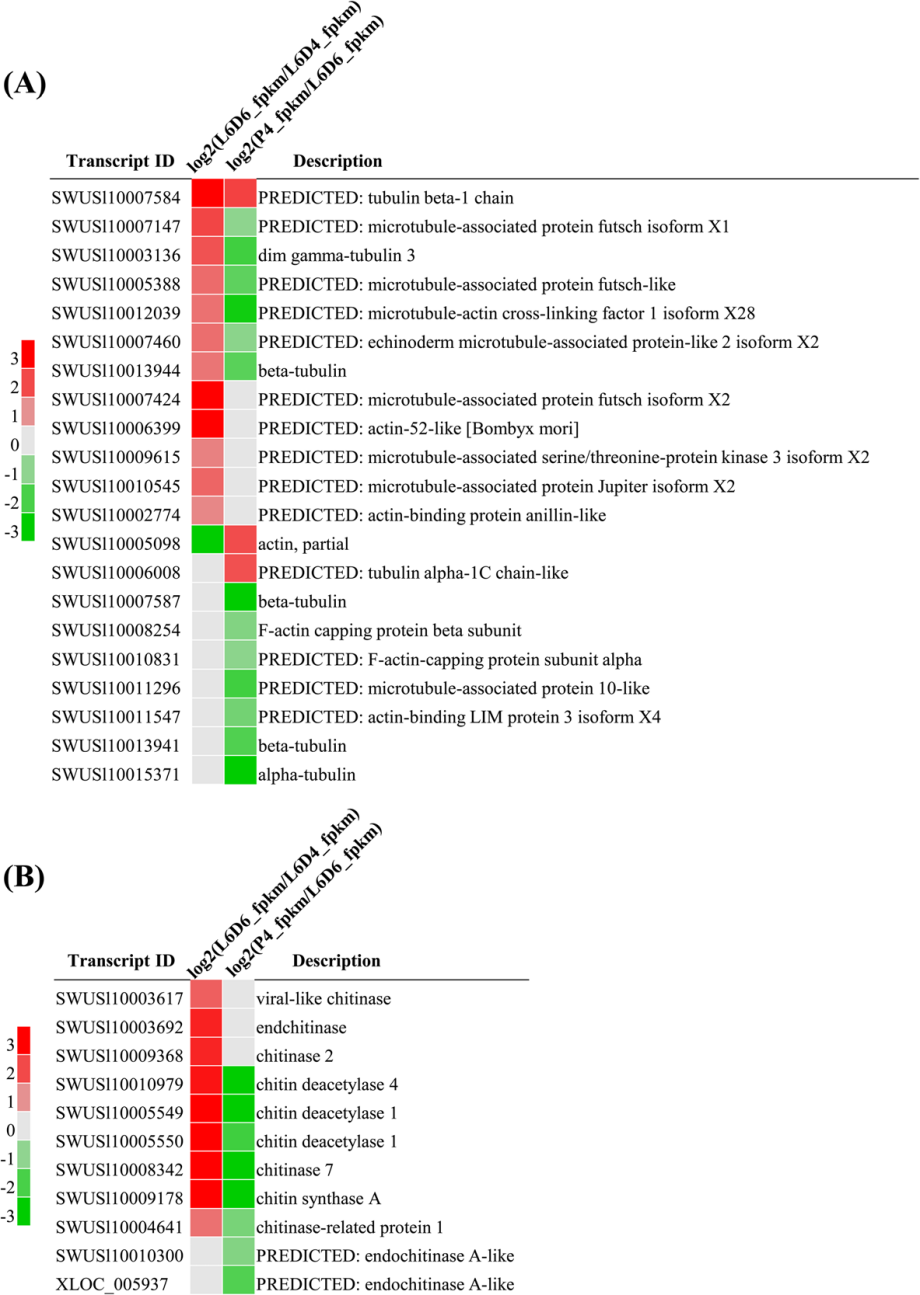
Expression patterns of transcripts related to cytoskeleton (**a**) and chitin metabolism **b**. The red represents the significantly up-regulated genes; the gray represents the genes not differentially expressed; the green represents the significantly down-regulated genes

The testicular fusion occurs during the larva-to-pupa metamorphosis, during which many of the tissues and organs are remodeled. The cytoskeletal proteins and insect chitin are involved in the remodeling of the tissue and organ [28, 29]. To study whether cytoskeleton and chitin are involved in testicular fusion, the expression of these genes was analyzed. The analysis found that the cytoskeletal proteins, including actin, tubulin, actin binding protein and microtubule-associated proteins were up-regulated from L6D4 to L6D6, and down-regulated from L6D6 to P4D (Fig. 6a). The expression pattern of chitinases, chitin deacetylases and chitin synthase A was similar to that of the cytoskeletal proteins (Fig. 6b). The results indicated that the testis fusion during the larva-to-pupa metamorphosis involved in testis remodeling through the roles of cytoskeletal proteins and chitin metabolism-related proteins.

### Expression of ECM component proteins

**Fig. 7:**
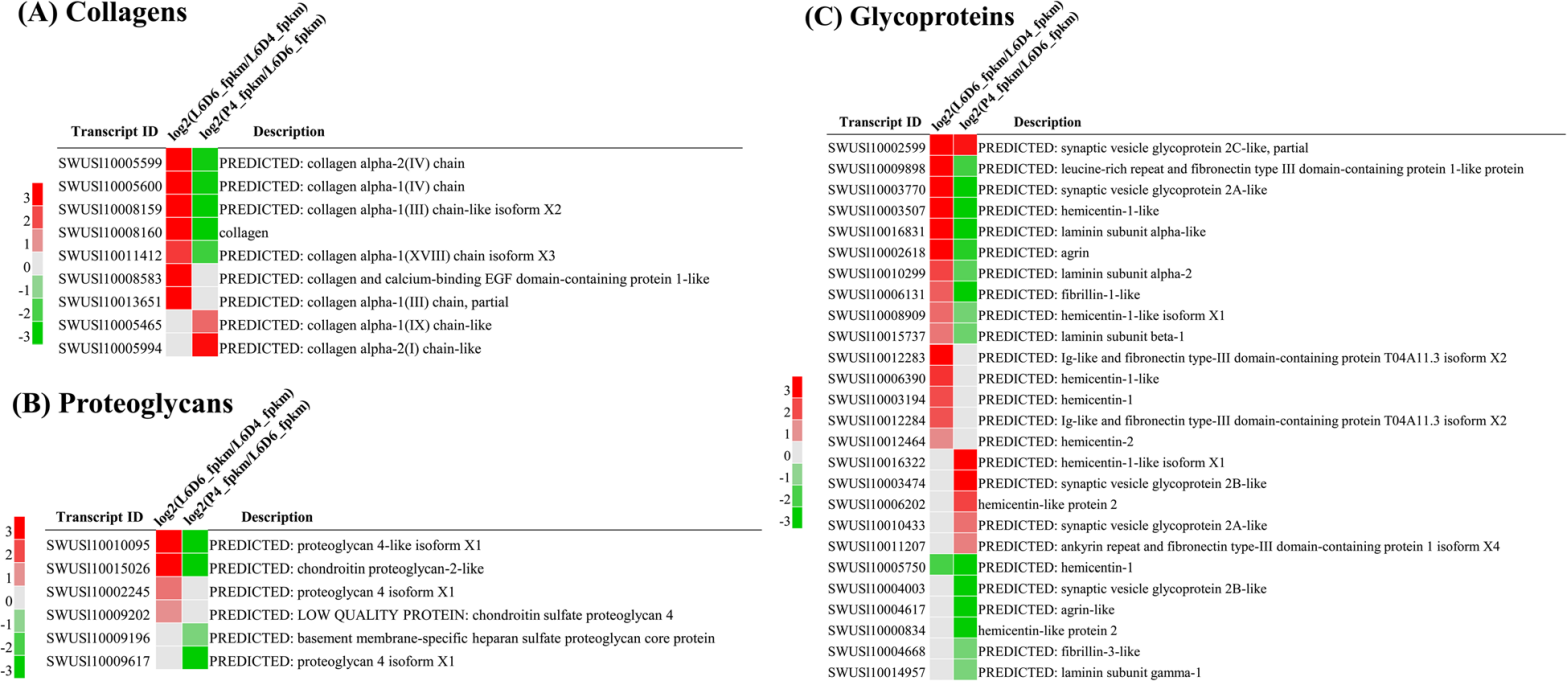
Expression patterns of the ECM component proteins. **a** collagens; **b** proteoglycans; **c** glycoproteins. The red represents the significantly up-regulated genes; the gray represents the genes not differentially expressed; the green represents the significantly down-regulated genes

**Fig. 8:**
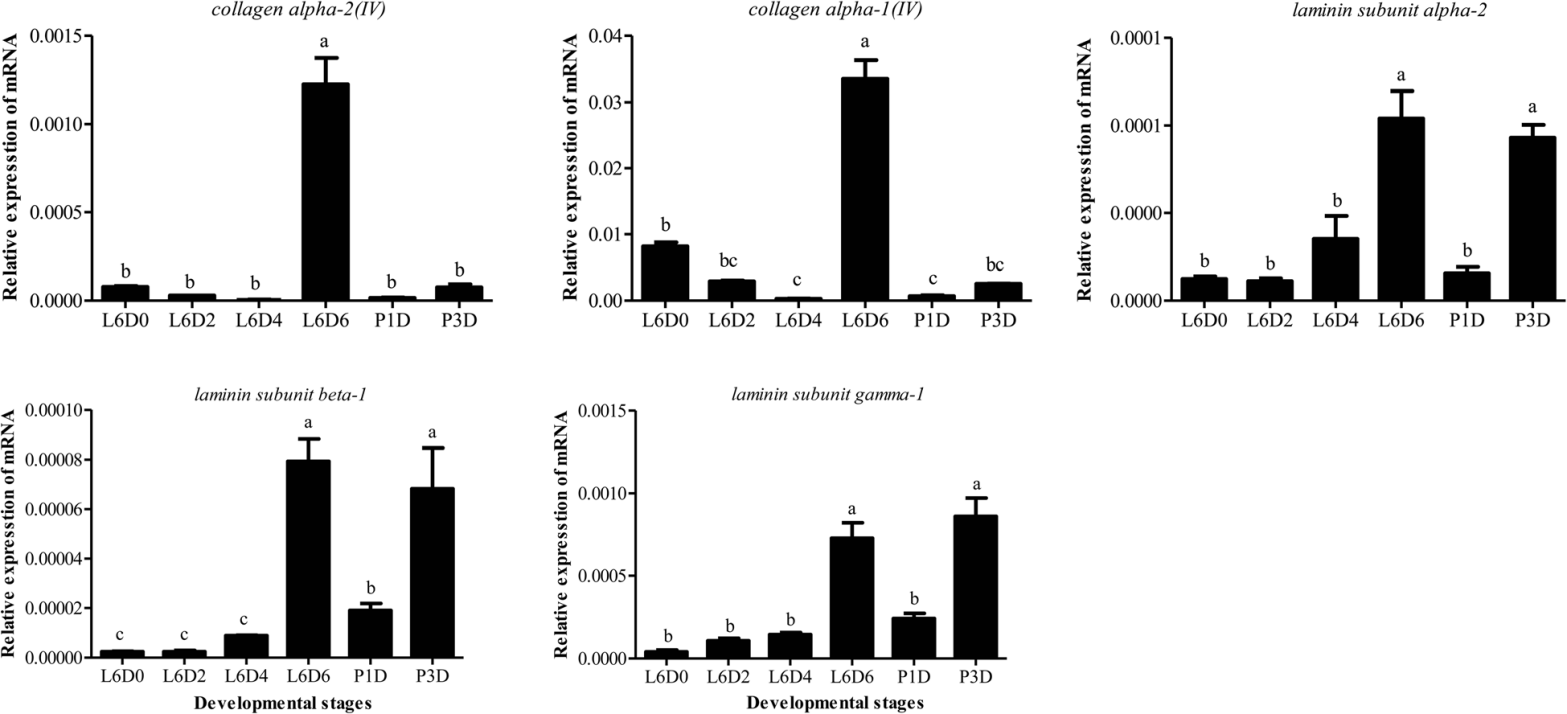
qRT-PCR analysis of the expression of the selected ECM component protein genes in the testes at the six different developmental stages in X-axis. The two collagen genes were *collagen alpha-1(IV)* and *collagen alpha-2(IV)* in Fig. 7a. The three glycoprotein laminin genes were l*aminin subunit alpha-2*; *laminin subunit beta-1*; *laminin subunit gamma-1* in Fig. 7b. The method and other information are the same as the Fig. 4. The significance is represented with different letters

ECM proteins usually consist of collagens, proteoglycans and glycoproteins and these proteins function in physical support for tissue integrity and elasticity, as well as the microenvironment of a cell, which influences cell behaviors, such as cell proliferation, adhesion and migration [30]. GO and KEGG analysis of DEG genes indicated those genes related to cell, cell part, membrance and membrance part and ECM-receptor interaction pathway were up-regulated when the testes were fusing and down-regulated after the testicular fusion. so, we are interested in these DEGs (Additional file 3). To study the roles of the ECM components in the testicular fusion, the expression of collagens, proteoglycans and glycoproteins were analyzed. Most of these genes were up-regulated at L6D6, when the testicular fusion occurred (Fig. 7). Two collagen genes (SWUSl10005600, XM_022978189.1, collagen alpha-1(IV) chain; SWUSl10005599, XM_022978190.1, collagen alpha-2(IV) chain) and the three glycoprotein laminin genes (SWUSl10014957, XM_022976705.1, laminin subunit gamma-1; SWUSl10015737, XM_022973420.1, laminin subunit beta-1; and SWUSl10010299, XM_022964712.1, laminin subunit alpha-2) were selected for qRT-PCR verification (Fig. 8), the results revealed that ECM component proteins may be involved in the testicular fusion in *S. litura*.

### Expression of ECM-associated proteins: mucins and lectins

**Fig. 9:**
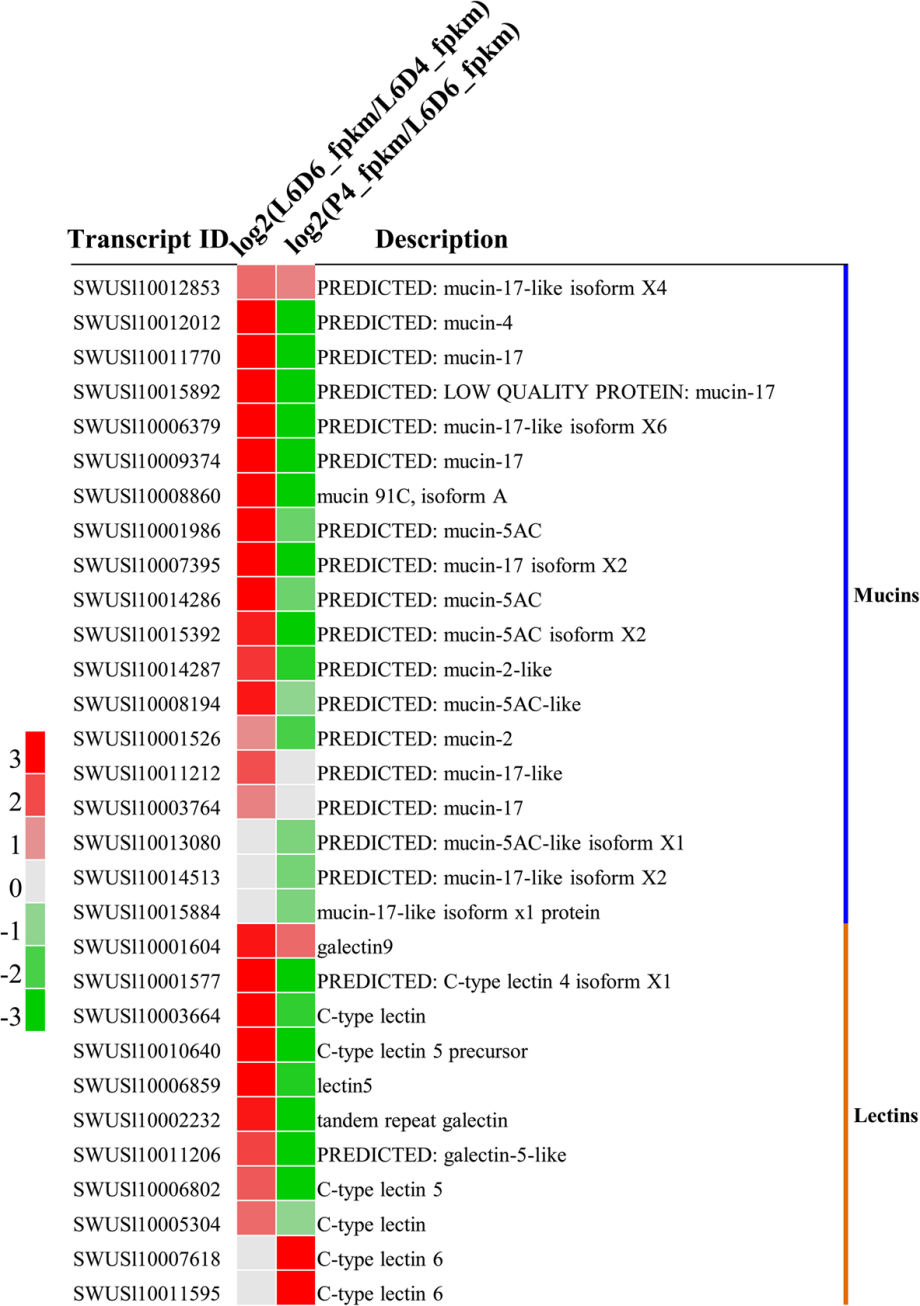
Expression patterns of the ECM-associated proteins: mucins and lecins. The red represents the significantly up-regulated genes; the gray represents the genes not differentially expressed; the green represents the significantly down-regulated genes

In this study, ECM-associated proteins: mucins and lectins were analyzed, these genes were up-regulated significantly during the testicular fusion (from L6D4 to L6D6), and down-regulated after the testicular fusion (from L6D6 to P4D) (Fig. 9). The expression patterns of these genes were consistent with that of the ECM component proteins, implying that the ECM-associated proteins and ECM component proteins may work together in the testicular fusion in *S. litura*.

### Expression of ECM receptor integrins

**Fig. 10:**
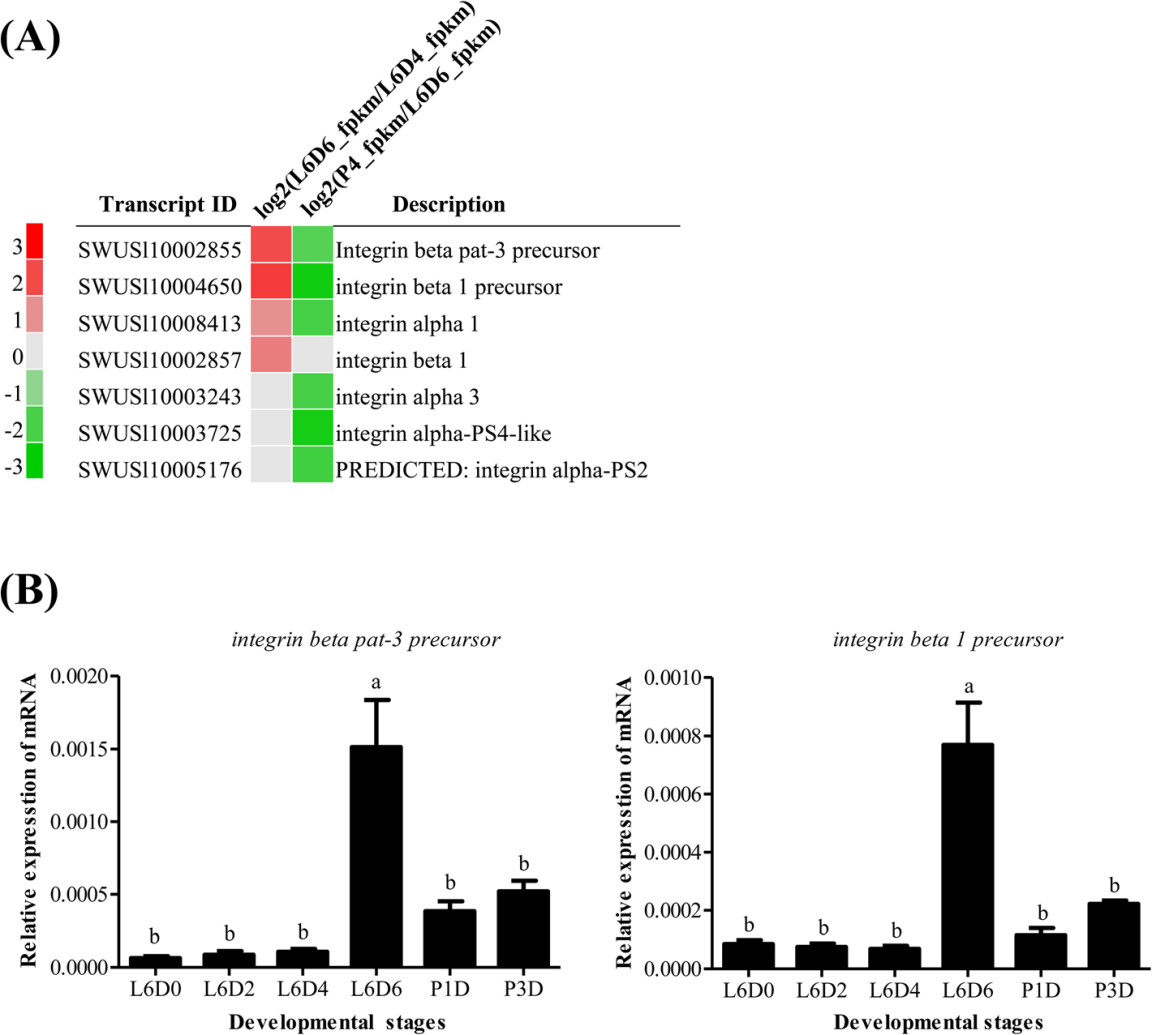
Expression of the *integrin* genes. **a** Transcriptomic analysis of the *integrin* expression; **b** qRT-PCR analysis of the *integrin* expression. The method and other information are the same as the Fig. 4. The significance is represented with different letters

The ECM-receptor interaction plays important roles in controlling cytoskeletal dynamics and regulating diverse functions including cell survival, differentiation, migration, attachment and focal adhesion assembly [28]. In this study, the ECM receptors integrins were significantly up-regulated at L6D6 as detected by both transcriptome and qRT-PCR analyses (Fig. 10). These results implied that all of the ECM component proteins, their receptor integrins and ECM-related proteins may jointly participate in the testicular fusion in *S. litura*.

### Expression and function analysis of ECM-remodeling enzymes: matrix metalloproteinases (MMPs)

**Fig. 11:**
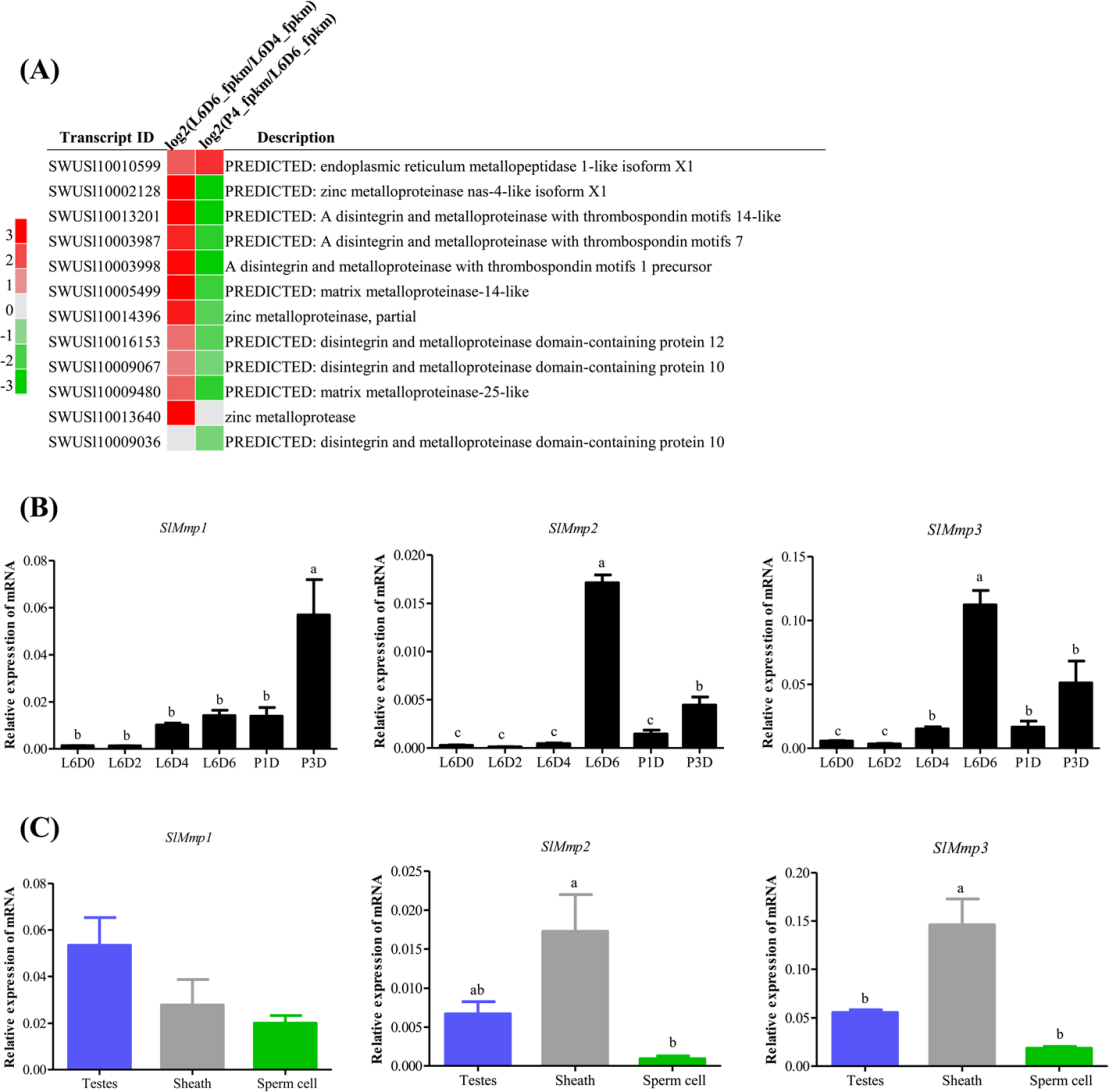
Developmental and spatial expression of *SlMmps* in the *S. litura* testes. **a** Transcriptomic analysis of the ECM-remodeling enzymes. The red represents the significantly up-regulated genes; the gray represents the genes not differentially expressed; the green represents the significantly down-regulated. **b** qRT-PCR analysis of the expression of the *SlMmp* genes in the testes at the six different developmental stages. *SlMmp1*: SWUSl10007900; *SlMmp2*:SWUSl10005499; *SlMmp3*:SWUSl10009480. **c** Expression levels of the *SlMmp* genes at whole testes, sheath and sperm cells. The method and other information are the same as the Fig. 4. The significance is represented with different letters

In this study, the genes related to the ECM remodeling were found and analyzed, MMPs and ADAMs were up-regulated significantly at L6D6 (Fig. 11a). To study the function of ECM-remodeling enzymes, MMPs were selected because three MMP transcripts were found in the testes (SWUSl10007900, XM_022966048.1, matrix metalloproteinase-14 isoform X3; SWUSl10005499, XM_022958284.1, matrix metalloproteinase-2-like and SWUSl10009480, XM_022964227.1, matrix metalloproteinase-2-like). Two *SlMmps* were significantly up-regulated during the testicular fusion process (Fig. 11b). Phylogenetic tree analysis was performed for the three MMP proteins. These three MMP proteins of *S. litura* were highly homologous to the *Bombyx mori* MMP proteins, BmMMP1, BmMMP2 and BmMMP3, respectively (Additional file 4). Thus, MMP SWUSl10007900, SWUSl10005499 and SWUSl10009480 were respectively named SlMMP1, SlMMP2 and SlMMP3 in this study.

To study the relationships between SlMMP proteins and the testicular fusion, the expression patterns of *SlMmps* were tested by qRT-PCR, the results indicated that *SlMmp1* was not differentially expressed, while *SlMmp2* and *SlMmp3* were up-regulated significantly during the testicular fusion (Fig. 11b), which was consistent with the transcriptomic analysis. To study the location of *SlMmps* RNA in the testis, the peritoneal sheath and sperm cells were isolated from the testis, the results indicated that *SlMmp2* and *SlMmp3* had a higher expression level in the peritoneal sheath than in sperm cells (Fig. 11c), while *SlMmp1* was not significantly differentially expressed in the peritoneal sheath and sperm cells (Fig. 11c). These results implied that SlMMP1 may not be related to the testicular fusion, whereas SlMMP2 and SlMMP3 may play roles in the peritoneal sheath helpful for testicular fusion.

**Fig. 12:**
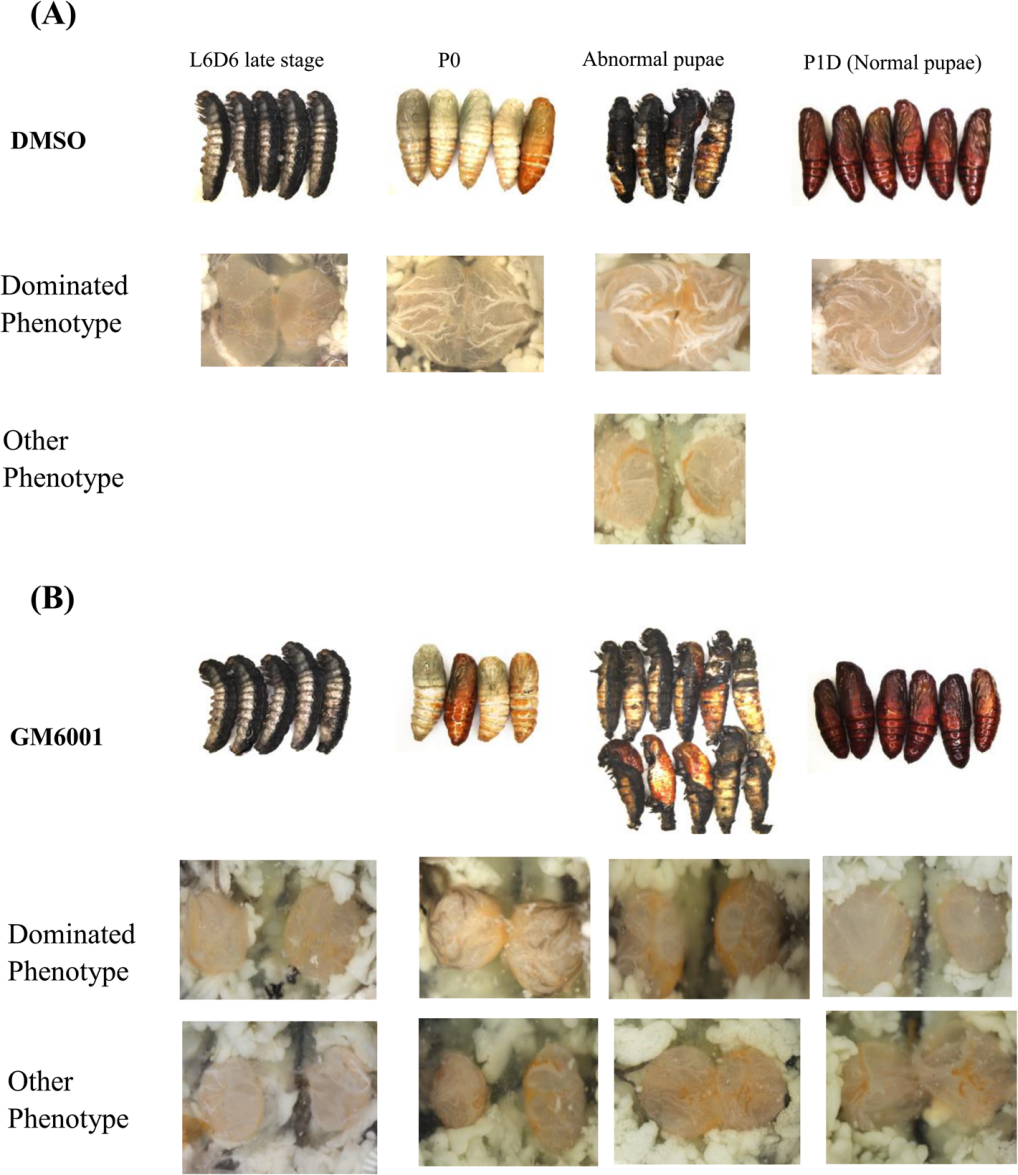
Phenotypic observation after the treatment with the MMP protein inhibitor GM6001. Phenotypic of male larvae, pupae and their testes of *S. litura* from L6D6 middle stage to P1D in the control group and the treatment group. The male larvae were injected with DMSO (**a**) or MMP protein inhibitor GM6001 (**b**) at L6D6 early stage. Twenty four hours later, in DMSO control group, most larvae developed into normal pupae and the testes were fused, a few larvae did not develop into normal pupae and was not able to shed during the larva-to-pupa metamorphosis. Most testes were fused for these melanism pupae (**a**). In the GM6001 treatment group, most larvae were not able to develop into normal pupae, and their testes did not fuse for both melanism and normal pupae (**b**). The 60–70 male larvae were injected for each treatment. Experiment was repeated three times. One representative result was shown

To study the function of the SlMMPs during the testicular fusion, a broad-spectrum inhibitor, GM6001, which is found to inhibit MMP function in some studies [31, 32], was applied to inhibit the SlMMPs action. GM6001 was injected into the 5th abdominal segment near the testis at the early L6D6 stage and then the testicular fusion process was statistically analyzed at the middle L6D6, late L6D6 stage, white pupae stage (P0) and 1-day-old pupae stage (P1D) (Fig. 12 and Table 1). The results indicated that almost half of larvae were not able to develop into normal pupae in the GM6001 treatment group, as compared to the control DMSO, in which most larvae developed into normal pupae. In all of the normal control pupae, the testis was able to get to close, adhere and fuse, however the fusion did not happen in the normal treatment pupae. For melanism pupae, the testis was fused for most control one while fusion did not happen for most treatment individuals (Table 1). Thus, these results implied that SlMMPs play a vital role in the testicular fusion.

**Table 1:** Statistics of the four kinds of testes (pre-fusion, slight fusion, fusion, torsion) after GM6001 treatment

			Pre-fusion	Slight fusion	Fusion	Torsion
			
Stages	Treatments	NO.				
Mid-L6D6	DMSO	10	2 (20%)	8 (80%)		
	GM6001	10	10 (100%			
Late-L6D6	DMSO	20	3 (15%)	7 (35%)	10 (50%)	
	GM6001	20	18 (90%)	2 (10%)		
P0	DMSO	10		2 (20%)	8 (80%)	
	GM6001	10	9 (90%)		1 (10%)	
P1D	DMSO (Normal pupae)	6				6 (100%)
	DMSO (Abnormal pupae)	7	1 (14.29%)	4 (57.12%)	2 (28.57%)	
	GM6001 (Normal pupae)	6	5 (83.33%)	1 (16.67%)		
	GM6001 (Abnormal pupae)	11	7 (63.64%)	4 (36.37%)		

*Mid-L6D6* Middle stage of L6D6, *Late-L6D6* Late stage of L6D6, *P0* Pupae, *P1D* 1-day-old pupae

## Discussion

The insect metamorphosis process is regulated coordinately by 20E and JH signals. The 20E signal related genes *BR-C*, *E75*, *E74*, *FTZ-F1* and *HR38* are up-regulated during the larva-to-pupa metamorphosis [33], contributing to the organ development such as the silk gland and wing disc in *B. mori* [34, 35]. 20E signal related genes were up-regulated duing the testicular fusion, which is speculated to be involved in the testis remodeling. In addition, it was also reported that the 20E stimulated the sperm development in *Manduca sexta* and European corn, *Ostrinia nubial*is during the metamorphosis [36, 37] and regulated the stem cell maintenance in *Drosophila* testis [38] In this study, 20E signal appeared to be more active than JH signal during the testicular fusion, implying that 20E signal may be involved in regulating the testicular fusion in *S. litura*.

By analyzing transcription factors in the transcriptome data during testicular fusion, 12 transcription factors were found to be highly expressed at L6D6 (Fig. 5). The gene *Ken* regulates the terminalia development of *Drosophila* [39] and is autonomously required for the self-renewal of somatic cyst stem cells in the *Drosophila* testis [40]. It is speculated that *ken* play similar roles during the testis fusion in *S. litura*. Chorion specific C/EBP factor is firstly isolated from *B. mori* follicular cells and characterized in lepidopteran insects and able to recognize homologous binding sites in the *chorion* gene promoters in flies and other moths [41]. The transcription factor βFTZ-F1 is very important in regulating the development of tissues and organs at the prepupal-pupal transition, such as wing [42], leg [43] and muscle [44] and is involved in the regulation of the cuticle protein genes [45]. In this study, a large number of cuticle protein genes were found from L6D4 to P4D stages (Additional file 5). Whether βFTZ-F1 is involved in the regulation of cuticle protein in the testes of *S. litura* should be further investigated*. Dfos/kayak* gene has been identified as a key regulator of epithelial cell morphogenesis during dorsal closure of the embryo and fusion of the adult thorax in *Drosophila* [46]. It may be also possible that *kayak* plays a role in cell morphogenesis and migration during the testicular fusion. The *Ets* gene is reported to regulate the tracheal cell migration and germ cell development [47, 48]. In insects, the testes are surrounded by branches of the trachea extended from the fifth or sixth abdominal segment (Fig. 1) [7]. Some studies report that the transcription factors *trachealess*, *spalt* and *Elbow* regulate the trachea development in *Drosophila* [49–51]. Thus, it is speculated that the above transcription factors, which were significantly up-regulated during the testis fusion at L6D6, probably are involved in regulating the testicular fusion events in *S. litura*.

The mechanisms of the cell or tissue fusion have been investigated in the past years which are complicated in different organisms [16–22]. There are similarities and differences in different fusion events. The genes that are related to cell recognition, migration and adhesion, such as the integrin, cadherin and ECM remodeling genes, are involved in different cells or myoblast fusions in flies and mice [16–22]. In this study, the cytoskeletal proteins are highly expressed when the testes began to fuse, which is similar to the myoblast fusion events and cellular fusion in the wing development of flies [22, 28], where the cytoskeletal protein actin plays important roles in cell shape and migration.

The ECM proteins including collagens, proteoglycans and glycoproteins bind with each other or with cell adhesion receptors forming a complex network around the cells residing in all tissues and organs [30]. It has been reported that collagen IV and the laminin glycoprotein are critical in providing the cell adhesion and stabilizing the overall structure [52]. For example, Collagen IV play a vital role in mediating inter-adipocyte adhesion in *Drosophila* [53], the loss of laminin function caused the failture of adhesion of wing-specific cells and formation of the wing blistering in *Bombyx mori* [54]. In this study, the ECM proteins were up-regulated when the testes fused (Fig. 7), implying that ECM components may be involved in cell adhesion during the testicular fusion in *S. litura*.

The integrin family is a kind of transmembrane receptors, of which the extracellular domains can bind ECM ligands [28]. Cell adhesion to the ECM is mediated via integrin receptors that regulates the bi-directional signaling, which is important for cell survival, proliferation, differentiation, migration and adhesion [21, 28, 55]. Integrins are involved in cell adhesion and fusion during the sperm-egg fusion [16–20], myoblast fusion [17, 21], cell-cell fusion in the heart development [19], stable adhesion between epithelia in wing development of insects [54] and cell migration in the nervous system development in mammals [56], In this study, the ECM components and its receptors integrins were also up-regulated at L6D6 (Fig. 7, Fig. 10), suggesting that the ECM-integrin interaction pathway may be involved in the fusion of testes by signaling cell migration and adhesion in *S. litura*.

The ECM is a highly dynamic structure that is constantly undergoing a remodeling involving in quantitative and qualitative changes in the ECM components. The ECM degradation is mediated by specific enzymes, such as MMPs and ADAMs. However, MMPs are the main enzymes involved in all ECM degradation and play crucial roles in the morphogenesis of tissues and organs [57, 58]. The ECM was degraded by MMPs for the neural crest cell migration during epithelial fusion in neural tube morphogenesis [59, 60]. During *Drosophila* heart development, MMPs regulated ECM differentiation and remodeling which promoted leading edge membrane dynamics of cardioblasts during cell migration [19]. In this study, MMPs and ADAMs were significantly up-regulated when two testes began to fuse (Fig. 11a, b) and *SlMmp2* and *SlMmp3* were highly expressed in peritoneal sheath (Fig. 11c). The broad spectrum MMP inhibitor GM6001 inhibited the testicular fusion process (Fig. 12, Table 1). It is reasonable to speculate that SlMMPs may play an important role for testicular fusion of *S. litura* by remodeling ECM of peritoneal sheath, It is similar to the cell fusion in the *Drosophila* heart development. Functional studies by CRISRP/Cas9 are currently performed to verify the function of those potential proteins.

## Conclusions

In this study, 12,339 transcripts were totally expressed from the testes at L6D4, L6D6 and P4D stages. From L6D4 to L6D6, 1676 transcripts were differentially expressed, while 3365 transcripts were differentially expressed from L6D6 to P4D. More transcripts were significantly up-regulated at L6D6 during the testicular fusion. 20E signal and some transcription factors which were specific highly expressed at L6D6 may be involved in the testicular fusion process. The cytoskeleton proteins, the proteins in the ECM-integrin interaction pathway, the ECM related proteins and the ECM-modifying enzymes MMPs may be involved in the cell shaping, migrating and adhering during the fusion of the testis.

## Methods

### Experimental animals and RNA extraction

*litura* larvae were provided by the Entomology Institute of SUN YAT-SEN University, Guangzhou, China and maintained in the Institute of Insect Science and Technology, South China Normal University. The larvae were reared at 25 °C on artificial diet under the condition of 12 h light and 12 h dark after egg hatching. The total number of *S. litura* larvae used in present study was around 2000. For RNA extraction, the testes were dissected from more than 10 male individuals at L6D4, L6D6 and P4D stages and usually there were three repeats for each stage for one experiment. Total RNA was extracted using the TRIzol reagent according to the manufacturer’s protocol (Invitrogen, California, USA).

### RNA-sequence, sequence mapping and annotation

About 5 μg RNA from L6D4, L6D6 and P4D testes was prepared. RNA-seq libraries were sequenced using the Illumina Hiseq™ 2000 platform by BGI company (Shenzhen, China). The clean reads of each sample were aligned to reference *S. litura* genome by TopHat2 software [25]. The assemble results were obtained from the TopHat2 alignment results by Cufflinks software [24]. The transcripts were annotated using the available protein databases including Nr (non-redundant protein databases), SwissProt, COG (Cluster of Orthologous Groups), and KEGG (Kyoto Encyclopedia of Genes Genomes) (e-value< 10^− 5^).

### Quality assessment of RNA-seq sequences

Raw reads (raw data) were saved in FASTQ format [61], and have been published on the website NCBI (https://www.ncbi.nlm.nih.gov/Traces/study/?acc=PRJNA447976). The reads containing adaptor sequences, unknown bases ‘NNN...’, and low quality reads were removed. The Q20, Q30, GC content are listed in the Additional file 1.

### The analysis of DEGs

The expression levels of genes were calculated by FPKM values, which were adjusted the number of fragments by the total number of the mapped fragments and the length of the gene [24, 26]. DEGs were obtained by using FPKM value of the log_2_**ǀ**fold changes > 1 and the standard of false discovery rate (FDR) < 0.001.

### Quantitative real-time PCR (qRT-PCR)

Two μg RNA from each sample were used to synthesize cDNA as the templates for qRT-PCR. The specific primers were designed based on the sequences aligned to *S. litura* genome (Additional file 6). The qRT-PCR experiments were performed using the QuantStudio™ 6 Flex Real-Time system (ABI, Life Technologies, Carlsbad USA) with SYBR® Select Master Mix (ABI). The total volume of qRT-PCR reactions was 20 μl, including 10 μl of 2 x SYBR® Select Master Mix (ABI), 0.8 μl of specific forward and reverse primers (10 μM). The cycling parameters were 50 °C for 2 min, 95 °C for 2 min, and followed by 40 cycles of 95 °C for 15 s, 60 °C for 1 min. The reference gene GAPDH (glyceraldehyde-3-phosphate dehydrogenase, LOC111366510) was used as a control gene. The relative expression levels were analyzed according to the method of 2^-ΔΔCt^ [62]. All experiment was repeated at least three times and one representing result was shown.

### Separation of peritoneal sheath and sperm cells

The testes were carefully isolated from L6D6 and put into the centrifuge tube and a little of PBS buffer was added. The testes were squeezed slightly with the tips, allowing the sperm cells released. A little amount of PBS buffer was used to wash the peritoneal sheath for five times, and the PBS buffer containing the sperm cells was collected in a new centrifuge tube. Then, the peritoneal sheaths were washed more twice with PBS to remove the sperm cells. The samples from 15 to 20 male individuals were used for RNA extraction. All experiment was repeated at least three times and one representing result was shown.

### Treatment of MMP inhibitor

The MMP inhibitor Ilomastat (Galardin; GM6001) (HY-15768, MedChemExpress) was selected. The GM6001 was dissolved in DMSO. The larvae at early L6D6 were injected with 3 μL inhibitor (20 μM/μL) around the testis in the 5th abdominal segment, and 60–70 male larvae per replicate were injected for each treatment. All experiment was repeated at least three times and one representing result was shown.

### Statistical analysis of data

For the genes expression analyses, One-way ANOVA analysis of variances was performed, followed by Turkey’s Multiple Comparison Test for significance analysis of the differences. Data were analyzed using the GraphPad Prism 5 software (Graphpad Software Inc., San Diego, CA). The values are mean ± SEM (Standard Error of Mean) (*n* = 3).

## Supplementary information

**Additional file 1. **Summary of the transcriptome data of the testis tissue in *S. litura*.

**Additional file 2. **Statistics of clean reads mapped to *S. litura* genome.

**Additional file 3.** GO and KEGG analysis of DEGs for L6D4 vs L6D6 and L6D6 vs PD3.

**Additional file 4.** Phylogenetic tree of the insect MMP proteins. Full-length protein sequences were obtained from the *S. litura* genome. Other MMPs protein sequences were downloaded from NCBI. All of MMPs protein sequences were aligned with ClustalW by the neighbor-joining method and the tree was built by using MEGA6. The *S. litura* MMPs are labeled with diamonds.

**Additional file 5.** Expression levels of cuticle protein genes during testicular fusion.

**Additional file 6.** Primers for qRT-PCR.

## Data Availability

All the data supporting the results in this article are included in the present and the Additional files. The RNA-seq raw read data have been submitted in the Sequence Read Archive of the NCBI (accession number: PRJNA447976).

## Abbreviations

20E: 20-hydroxyecdysone
BR-C: Broad complex
ECM: Extracellular matrix
EcR: Ecdysone receptor
FDR: False Discovery Rate
FPKM: Fragment Per kb per Million reads
JH: Juvenile hormone
Kr-h1: Krüppel homolog 1
L6D4: 4-day-old sixth instar larvae
L6D6: 6-day-old sixth instar larvae
Late-L6D6: Late stage of L6D6
Met: Methoprene-tolerant
Mid-L6D6: Middle stage of L6D6
MMP: Matrix metalloproteinase
P0: Pupae
P1D: 1-day-old pupae
P4D: 4-day-old pupae
SRC: Steroid receptor coactivator
USP: Ultraspiracle
