# Supplementary material for: Transcriptomic analysis of the testicular fusion in Spodoptera litura
Source: BMC Genomics. 2020 Feb 19;21:171. doi: 10.1186/s12864-020-6494-3 (PMC7029529; doi:10.1186/s12864-020-6494-3)
Supplement: Supplementary file 1 — Additional file 1. Summary of the transcriptome data of the testis tissue in S. litura. [file 12864_2020_6494_MOESM1_ESM.docx]

**Additional file 1**

**Table S1: Summary of the transcriptome data of the testis tissue in *S. litura.***

| Sample | Clean Reads | HQ Clean Reads | low quality(%) | Q20(%) | Q30(%) | GC(%) |
| --- | --- | --- | --- | --- | --- | --- |
| L6D4 | 4794144 | 4780380 | 0.27% | 97.85% | 93.88% | 42.67% |
| L6D6 | 4907888 | 4891555 | 0.27% | 97.75% | 93.68% | 43.07% |
| P4D | 4870410 | 4854931 | 0.3%) | 97.76% | 93.61% | 42.76% |

Q20: percentage of every base with a Phred value of at least 20. Q30: percentage of every base with a Phred value of at least 30. GC: percentage of GC bases.
