# Supplementary material for: Transcriptomic analysis of the testicular fusion in Spodoptera litura
Source: BMC Genomics. 2020 Feb 19;21:171. doi: 10.1186/s12864-020-6494-3 (PMC7029529; doi:10.1186/s12864-020-6494-3)
Supplement: Supplementary file 2 — Additional file 2. Statistics of clean reads mapped to S. litura genome. [file 12864_2020_6494_MOESM2_ESM.docx]

**Additional file 2**

**Table S2: Statistics of clean reads mapped to *S. litura* genome.**

| Sample | Total Reads | Unmapped Reads | Unique Mapped Reads | Multiple Mapped reads | Mapping Ratio |
| --- | --- | --- | --- | --- | --- |
| L6D4 | 4777488 | 252790 | 4524698 | 0 (0%) | 94.71% |
| L6D6 | 4886356 | 284082 | 4602274 | 0 (0%) | 94.19% |
| P4D | 4852421 | 257828 | 4594593 | 0 (0%) | 94.69% |
