# Supplementary material for: Transcriptomic analysis of the testicular fusion in Spodoptera litura
Source: BMC Genomics. 2020 Feb 19;21:171. doi: 10.1186/s12864-020-6494-3 (PMC7029529; doi:10.1186/s12864-020-6494-3)
Supplement: Supplementary file 3 — Additional file 3. GO and KEGG analysis of DEGs for L6D4 vs L6D6 and L6D6 vs PD3. [file 12864_2020_6494_MOESM3_ESM.docx]

Additional file 3

Figure S1(related to Fig. 7)


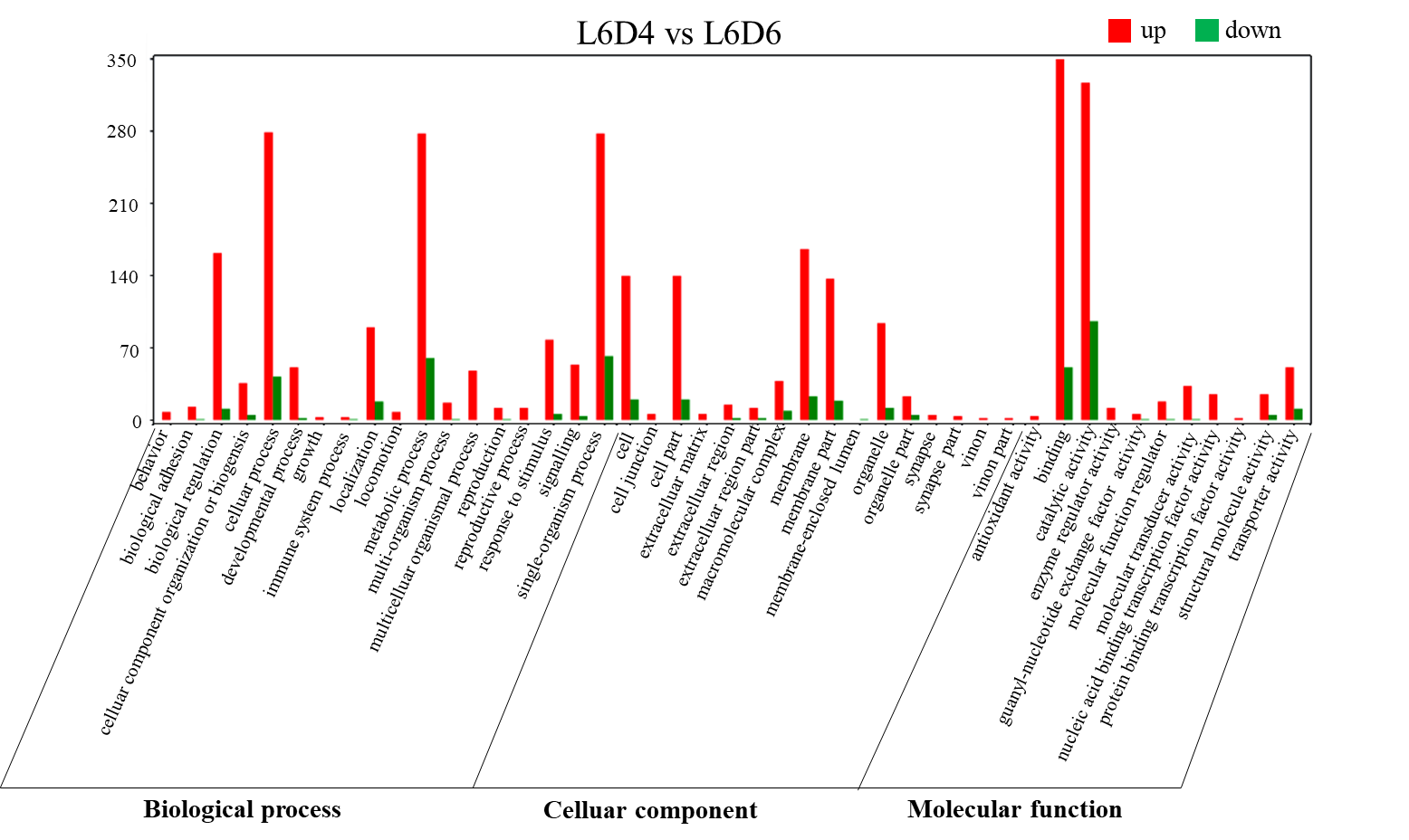


A


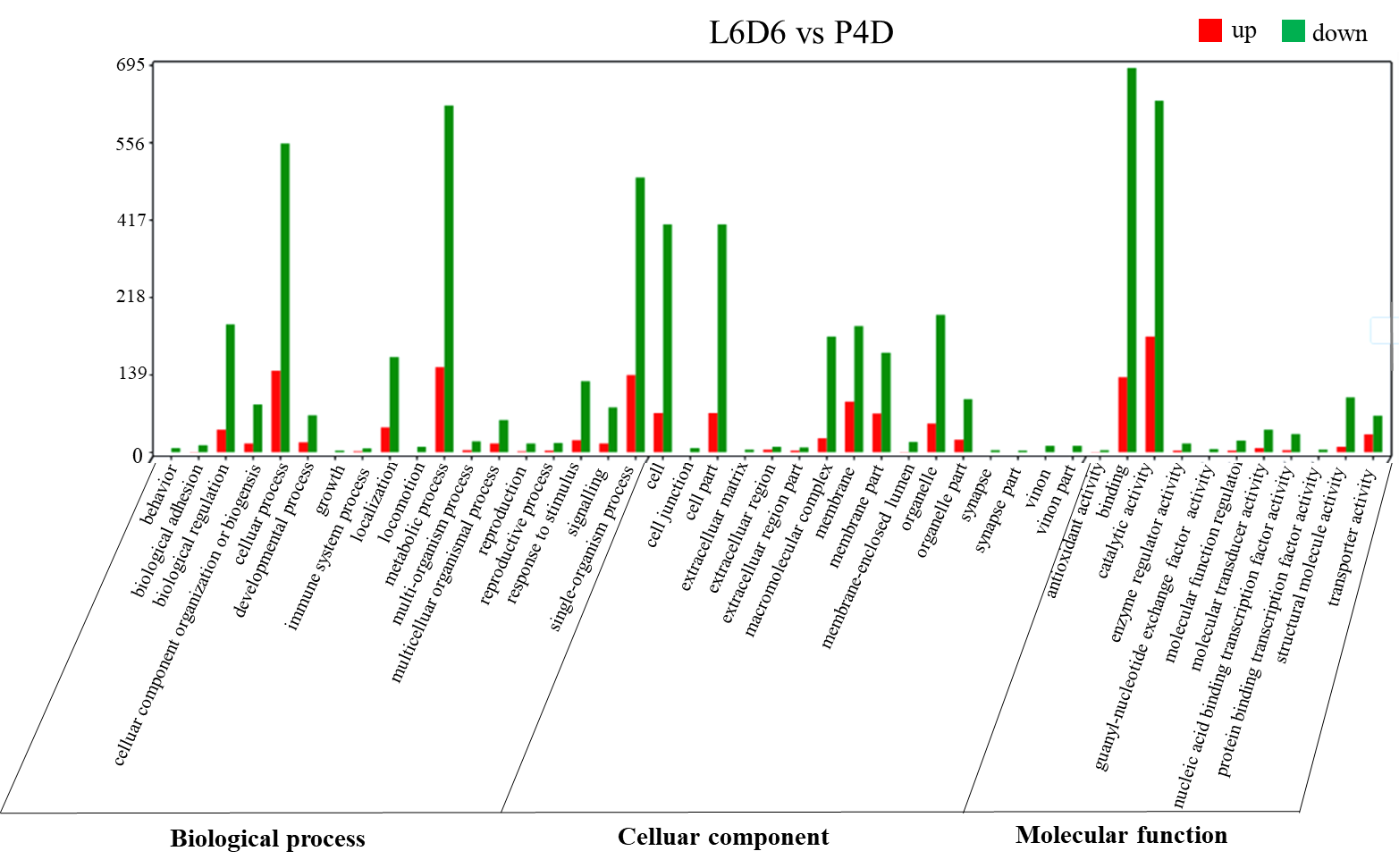


B


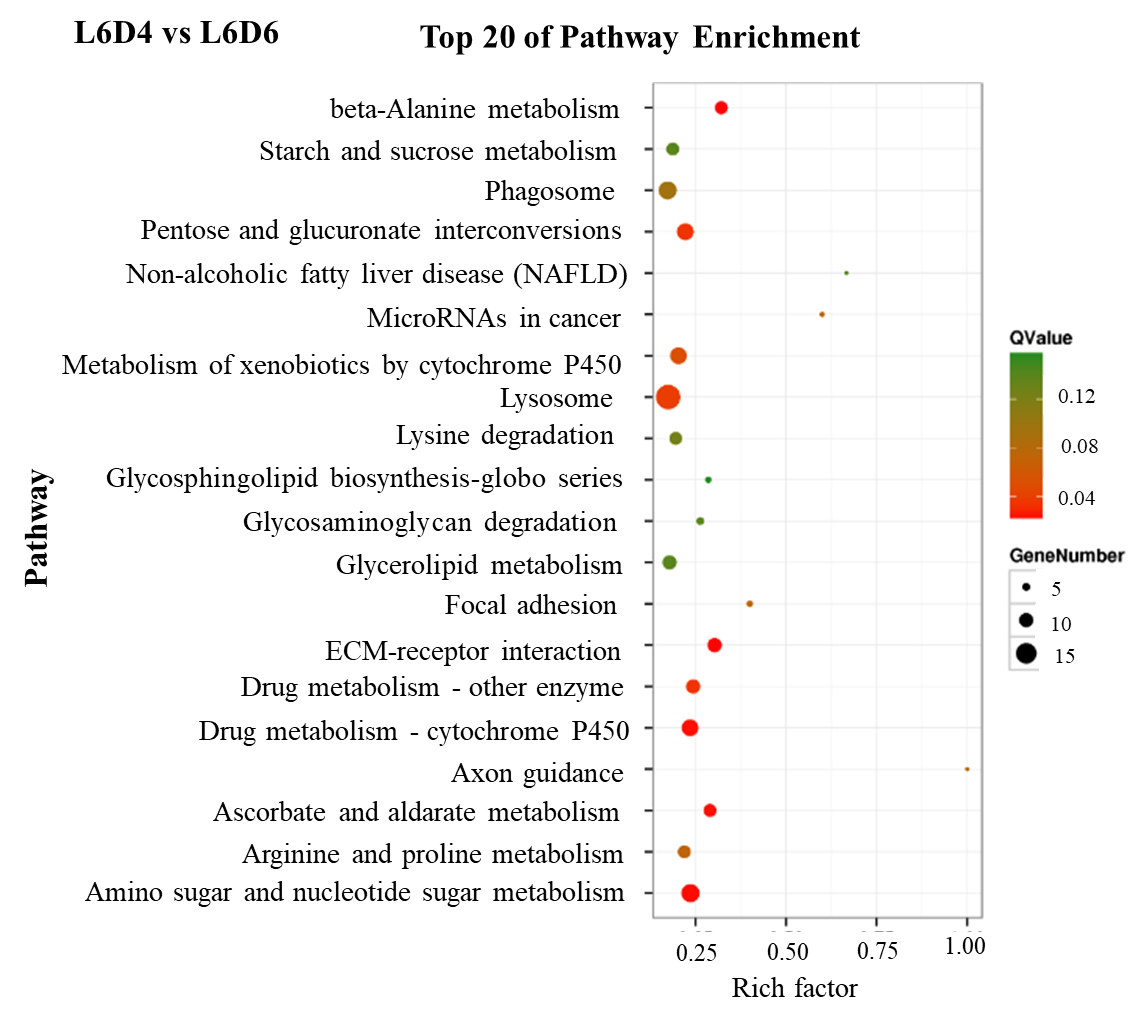


C


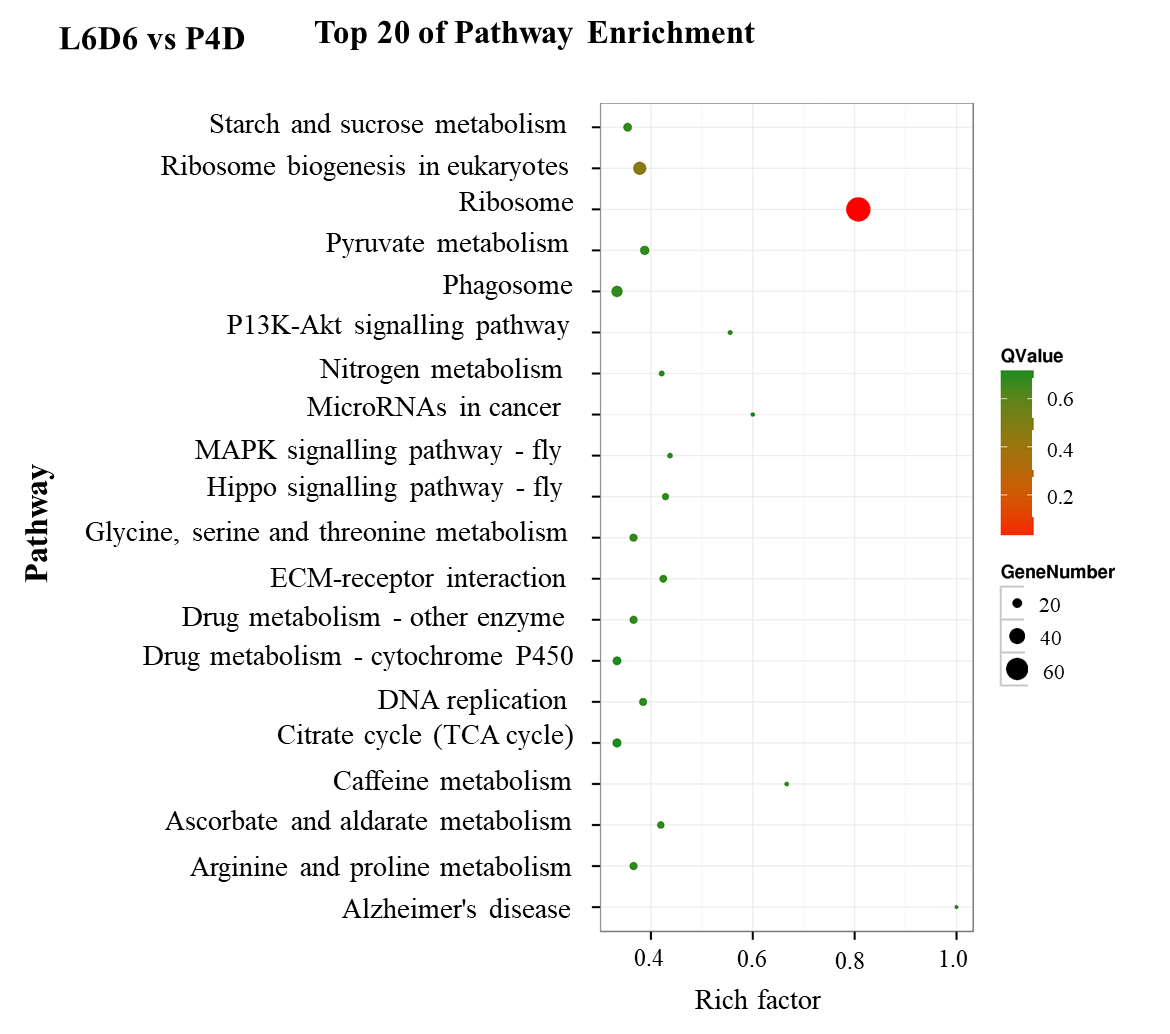


D

Fig. S1. GO analysis of the DEGs of L6D4 vs L6D6 (A), and L6D6 vs P4D (B). KEGG analysis top 20 of enriched pathway for DEGs of L6D4 vs L6D6 (C) and L6D6 vs P4D (D).
