# Supplementary material for: Transcriptomic analysis of the testicular fusion in Spodoptera litura
Source: BMC Genomics. 2020 Feb 19;21:171. doi: 10.1186/s12864-020-6494-3 (PMC7029529; doi:10.1186/s12864-020-6494-3)
Supplement: Supplementary file 4 — Additional file 4. Phylogenetic tree of the insect MMP proteins. Full-length protein sequences were obtained from the S. litura genome. Other MMPs protein sequences were downloaded from NCBI. All of MMPs protein sequences were aligned with ClustalW by the neighbor-joining method and the tree was built by using MEGA6. The S. litura MMPs are labeled with diamonds. [file 12864_2020_6494_MOESM4_ESM.docx]

**Additional file 4**

**Figure S2 (related to Fig. 11)**

**Figure S2. Phylogenetic tree of the insect MMP proteins.** Full-length protein sequences were obtained from the *S. litura* genome. Other MMPs protein sequences were downloaded from NCBI. All of MMPs protein sequences were aligned with ClustalW by the neighbor-joining method and the tree was built by using MEGA6. The *S. litura* MMPs are labeled with diamonds.
