# Supplementary material for: Transcriptomic analysis of the testicular fusion in Spodoptera litura
Source: BMC Genomics. 2020 Feb 19;21:171. doi: 10.1186/s12864-020-6494-3 (PMC7029529; doi:10.1186/s12864-020-6494-3)
Supplement: Supplementary file 5 — Additional file 5. Expression levels of cuticle protein genes during testicular fusion. [file 12864_2020_6494_MOESM5_ESM.docx]

**Additional file 5**

**Table S3: Expression levels of cuticle protein genes during testicular fusion.**

| ID | Description | L6D4-FPKM | L6D6-FPKM | P4D-FPKM |
| --- | --- | --- | --- | --- |
| SWUSl10011689 | PREDICTED: pupal cuticle protein C1B-like [Bombyx mori] | 0.001 | 347.19 | 0.001 |
| SWUSl10006211 | larval cuticle protein LCP-17 precursor [Bombyx mori] | 232.78 | 4.79 | 3.28 |
| SWUSl10010732 | larval cuticle protein 1 precursor [Bombyx mori] | 100.82 | 0.001 | 2.07 |
| SWUSl10010769 | cuticle protein [Helicoverpa armigera] | 0.6 | 12.02 | 4.31 |
| SWUSl10011683 | PREDICTED: pupal cuticle protein C1B-like [Bombyx mori] | 0.001 | 199.02 | 0.001 |
| SWUSl10011684 | PREDICTED: pupal cuticle protein C1B-like [Bombyx mori] | 0.001 | 242.87 | 0.001 |
| SWUSl10011685 | PREDICTED: pupal cuticle protein C1B-like [Bombyx mori] | 0.001 | 137.45 | 0.001 |
| SWUSl10011687 | PREDICTED: pupal cuticle protein C1B-like [Bombyx mori] | 0.001 | 915 | 2.75 |
| SWUSl10011688 | PREDICTED: pupal cuticle protein C1B-like [Bombyx mori] | 0.001 | 915 | 2.75 |
| SWUSl10011689 | PREDICTED: pupal cuticle protein C1B-like [Bombyx mori] | 0.001 | 347.19 | 0.001 |
| SWUSl10008079 | cuticle protein [Bombyx mori] | 0.001 | 5.18 | 0.001 |
| SWUSl10008080 | PREDICTED: cuticle protein 7-like [Plutella xylostella] | 0.001 | 1.36 | 0.001 |
| SWUSl10008109 | PREDICTED: cuticle protein 8-like [Plutella xylostella] | 2.7 | 0.001 | 2.7 |
| SWUSl10008270 | pupal cuticle protein [Heliothis virescens] | 0.75 | 2.24 | 0.001 |
| SWUSl10013761 | pupal cuticle protein precursor [Bombyx mori] | 0.001 | 1.77 | 0.001 |
| SWUSl10001028 | cuticular protein RR-2 motif 67 precursor [Bombyx mori] | 0.001 | 52.44 | 0.001 |
| SWUSl10001029 | cuticular protein RR-2 motif 70 [Danaus plexippus] | 0.001 | 124.8 | 0.001 |
| SWUSl10001030 | cuticular protein RR-2 motif 70 [Danaus plexippus] | 0.001 | 302.65 | 0.001 |
| SWUSl10001031 | cuticular protein RR-2 motif 69 precursor [Bombyx mori] | 3.12 | 1857.2 | 3.2 |
| SWUSl10001034 | cuticular protein PpolCPR67A [Papilio polytes] | 0.001 | 112.12 | 0.001 |
| SWUSl10002405 | cuticular protein PxutCPT4 [Papilio xuthus] | 0.001 | 1734.69 | 3.54 |
| SWUSl10004247 | cuticular protein RR-1 motif 56 precursor [Bombyx mori] | 0.001 | 10.79 | 0.001 |
| SWUSl10005421 | cuticular protein PpolCPR55 [Papilio polytes] | 0.001 | 1504.97 | 0.001 |
| SWUSl10005425 | cuticular protein hypothetical 28 precursor [Bombyx mori] | 0.001 | 666.19 | 0.84 |
| SWUSl10006738 | cuticular protein RR-1 motif 10 precursor [Bombyx mori] | 0.001 | 15.09 | 0.001 |
| SWUSl10007758 | cuticular protein PpolCPG24 [Papilio polytes] | 0.64 | 419.43 | 3.29 |
| SWUSl10008082 | cuticular protein CPR99 [Danaus plexippus] | 16.7 | 1.05 | 0.001 |
| SWUSl10008100 | cuticular protein PxutCPR123 [Papilio xuthus] | 0.001 | 111.73 | 8.21 |
| SWUSl10008107 | cuticular protein RR-2 motif 127 [Danaus plexippus] | 3.94 | 26.86 | 31.57 |
| SWUSl10008269 | cuticular protein hypothetical 2 precursor [Bombyx mori] | 0.001 | 53.86 | 0.001 |
| SWUSl10008271 | cuticular protein PpolCPH4A [Papilio polytes] | 0.001 | 108.27 | 0.001 |
| SWUSl10008451 | cuticular protein CPH42 [Papilio xuthus] | 0.001 | 1009.92 | 0.001 |
| SWUSl10009253 | cuticular protein CPG14 [Danaus plexippus] | 0.001 | 769.74 | 0.001 |
| SWUSl10010750 | cuticular protein RR-1 motif 34 precursor [Bombyx mori] | 23.3 | 94.46 | 37.47 |
| SWUSl10010751 | PREDICTED: cuticular protein RR-1 motif 33 isoform X1 [Bombyx mori] | 1.46 | 52.64 | 28.47 |
| SWUSl10010969 | cuticular protein RR-1 motif 21 precursor [Bombyx mori] | 0.001 | 19.52 | 0.8 |
| SWUSl10011300 | cuticular protein CPT2 [Papilio xuthus] | 0.001 | 1766.51 | 0.68 |
| SWUSl10011662 | cuticular protein hypothetical 7 precursor [Bombyx mori] | 0.001 | 6.21 | 0.001 |
| SWUSl10011676 | cuticular protein hypothetical 19 [Danaus plexippus] | 1.31 | 3094.04 | 4.04 |
| SWUSl10011679 | cuticular protein hypothetical 22 [Danaus plexippus] | 0.41 | 3173.55 | 0.001 |
| SWUSl10011680 | cuticular protein hypothetical 23 [Danaus plexippus] | 0.001 | 411 | 0.001 |
| SWUSl10012743 | cuticular protein hypothetical 33 precursor [Bombyx mori] | 0.001 | 80.35 | 0.001 |
| SWUSl10013174 | cuticular protein RR-1 motif 1 precursor [Bombyx mori] | 0.001 | 8.87 | 0.001 |
| SWUSl10014139 | cuticular protein RR-2 motif 143 [Bombyx mori] | 3.79 | 32.53 | 5.2 |
| SWUSl10014762 | cuticular protein RR-1 motif 47 [Danaus plexippus] | 35.86 | 9.5 | 0.001 |
| SWUSl10015395 | cuticular protein PpolCPG12 [Papilio polytes] | 0.001 | 63.99 | 1.21 |
| SWUSl10015397 | cuticular protein PpolCPG11 [Papilio polytes] | 0.001 | 99.39 | 0.001 |
| SWUSl10015583 | cuticular protein CPG8 [Papilio xuthus] | 0.001 | 6.69 | 0.001 |
| SWUSl10015585 | cuticular protein glycine-rich 9 precursor [Bombyx mori] | 0.001 | 1624.93 | 1.14 |
