# Supplementary material for: Transcriptomic analysis of the testicular fusion in Spodoptera litura
Source: BMC Genomics. 2020 Feb 19;21:171. doi: 10.1186/s12864-020-6494-3 (PMC7029529; doi:10.1186/s12864-020-6494-3)
Supplement: Supplementary file 6 — Additional file 6. Primers for qRT-PCR. [file 12864_2020_6494_MOESM6_ESM.docx]

**Additional file 6**

| **Table S3: Primers for qRT-PCR** | |
| --- | --- |
| Gene name | Sequence |
| *SlMmp*1-F (SWUSl10007900) | 5'-CGCTCTGTGGTGACCCTAAGTT-3' |
| *SlMmp*1-R (SWUSl10007900) | 5'-AAGTAAGTCTTGCCGTTTTTGTATG-3' |
| *SlMmp*2-F (SWUSl10009480) | 5'-GGAGGAAAGATACTGGCGGT-3' |
| *SlMmp*2-R (SWUSl10009480) | 5'-AGCAGCGTCCACAGGGTAAG-3' |
| *SlMmp*3-F (SWUSl10005499) | 5'-GAGAAGTGGAAGTACACCAACTTGA-3' |
| *SlMmp*3-R (SWUSl10005499) | 5'-AAACGCTTAGCAAAGGAAACGA-3' |
| *integrin beta*1-F (SWUSl10004650) | 5'-ACACTGGGAAGTTTACTGTCTGCTA-3' |
| *integrin beta*1-R (SWUSl10004650) | 5'-CTACAAATGAGCCGAATCCGA-3' |
| *integrin beta pat*3-F (SWUSl10002855) | 5'-GGAATCGAAGTCAACCCAGTATC-3' |
| *integrin beta pat*3-R (SWUSl10002855) | 5'-AGTCCCAACACATTCAGGCT-3' |
| *collagen(IV) alpha*1-F (SWUSl10005600) | 5'-GAAGAAACTCCTATGAATCCAGTGC-3' |
| *collagen(IV) alpha*1-R (SWUSl10005600) | 5'-GCATAACAAAACTGTATCCGATCCA-3' |
| *collagen(IV) alpha*2-F (SWUSl10005599) | 5'-TTGGAAGAACAATATACGTGCCTCC-3' |
| *collagen(IV) alpha*2-R (SWUSl10005599) | 5'-GGGCGTTTTCAGCGCGTATT-3' |
| *laminin alpha2*-F (SWUSl10010299) | 5'-AAAGGAGGCACGCCATCTAC-3' |
| *laminin alpha*2-R (SWUSl10010299) | 5'-CCAGCCGTCTGTATGTGTCT-3' |
| *laminin beta*1-F (SWUSl10015737) | 5'-TCGTGTGAATTAAGCTCCTGCTAC-3' |
| *laminin beta*1-R (SWUSl10015737) | 5'-CCCATTTTCAGATTGCCACC-3' |
| *laminin gamma*1-F (SWUSl10014957) | 5'-TTGCTTCAAGTCAGTTCCTGGG-3' |
| *laminin gamma*1-R (SWUSl10014957) | 5'-ATTGGTAGGTTCTGGATTGTTCTG-3' |
| *Slgapdh*-F(LOC111353761) | 5'-GTATGGCTTTCCGTGTTCCTGT-3' |
| *Slgapdh*-R(LOC111353761) | 5'-ACCTTCTGCTTGATAGCGTCGTA-3' |
| *class A basic helix-loop-helix protein 15-like* (*bHLH15*)*-*F (SWUSl10001165) | 5'-GAATGTAGAGCCTGTGTTCGTGTT-3' |
| *class A basic helix-loop-helix protein 15-like*(*bHLH15*)*-*R(SWUSl10001165) | 5'-GCTGGAGTCTTCTCGGTTCTGAT-3' |
| *Kayak*-F (SWUSl10002048) | 5'-CTACAAGAGGAGATTCGCAAACTAA-3' |
| *Kayak*-R (SWUSl10002048) | 5'-TTCAGGCATTTCTGGGTAGGA-3' |
| *CCAAT/enhancer-binding protein* (*C/EBP-γ*)-F (SWUSl10002784) | 5'-GACACATTCTCCCGAGTCAAGC-3' |
| *CCAAT/enhancer-binding protein* (*C/EBP-γ*)-R (SWUSl10002784) | 5'-CCAGCAGTTTCTCAAGGTCCAT-3' |
| *chorion specific C/EBP* (*C/EBP*) -F (SWUSl10004173) | 5'-CCAAGCAGCAGACACAGGACA-3' |
| *chorion specific C/EBP* (*C/EBP*) -R (SWUSl10004173) | 5'-CCAGCAGTTTCTCAAGGTCCAT-3' |
| *Ken1*-F (SWUSl10005742) | 5'-CCACCTCTCTATCAACGGCATC-3' |
| *Ken1*-R (SWUSl10005742) | 5'-ACATCCTCTAAGTCAGGGGGCT-3' |
| *Salm-X2*-F (SWUSl10009158) | 5'-CGCCGAGTCCTTCTCAATCA-3' |
| *Salm-X2*-R (SWUSl10009158) | 5'-TGTGGCATCATTTTCGTCAGG-3' |
| *Elbow*-F (SWUSl10010461) | 5'-CGTGCCTAACAGCGAGAGAAAA-3' |
| *Elbow*-R (SWUSl10010461) | 5'-AGTTGTTGACGCACTGCCTTTG-3' |
| *Ets*-F (SWUSl10011082) | 5'-CGAGACGGATTGCGGATAGT-3' |
| *Ets*-R (SWUSl10011082) | 5'-GCATCGTGTTTGTGGACTCTTG-3' |
| *betaFTZ-F1*-F (SWUSl10011290) | 5'-CACAGTATGCGACCAAACCGA-3' |
| *betaFTZ-F1*-R (SWUSl10011290) | 5'-TTTTGTCTATGTGGCAGGCTCG-3' |
| *zinc finger homeobox protein 3*-F (SWUSl10011373) | 5'-TTCCCTCGTTTTGACTTATGG-3' |
| *zinc finger homeobox protein 3*-R (SWUSl10011373) | 5'-AAGGGGAATCGGGTGGATAT-3' |
| *trachealess* (*Trh*)-F (SWUSl10012934) | 5'-TAACCAAGAGAGGATGCCATTTCA-3' |
| *trachealess* (*Trh*)-R (SWUSl10012934) | 5'-CTATGGGAAAAGGAATACTGCGG-3' |
| *GF22772*-F (SWUSl10015888) | 5'-GACAAACCTCCAATGAGACCTTAC-3' |
| *GF22772*-R (SWUSl10015888) | 5'-TCGCTCAAATCTTCAGGTAGGA-3' |
| *EcRA*-F (SWUSl10014730) | 5’- CCCAAATGGAAAAATAGGTCGT -3’ |
| *EcRA*-R (SWUSl10014730) | 5’- GACGAGACATAGCTCCTCTTGTT -3’ |
| *USP1*-F (SWUSl10000767) | 5’- ATGTCAGTGGCGAAGAAAGA -3’ |
| *USP1*-R (SWUSl100007670 | 5’-ATCCAGCGAACAGTCAACAG -3’ |
| *BR-C Z2*-F(SWUSl10014313) | 5'-GCTCGCTATGCGGGAAGGTT-3' |
| *BR-C Z2*-R(SWUSl10014313) | 5'-ATGTGGGTCATCAGGGAGTTGC-3' |
| *BR-C Z4*-F(SWUSl10014312) | 5'-CTCTGCCACAAGGTGTTCCGA-3' |
| *BR-C Z4*-R(SWUSl10014312) | 5'-TTAGTGTCGGGGCTGGTGGTC-3' |
| *Met1*-F(SWUSl10002734) | 5'-GCTGGGTCATTTGTGTGTTACG-3' |
| *Met1*-R(SWUSl10002734) | 5'-TTTCCAAAAATCCTCGTGTTCTC-3' |
| *Kr-h1*-F(SWUSl10007674) | 5'-ACACAAAAAAACAAATGGAGGCT-3' |
| *Kr-h1*-R(SWUSl10007674) | 5'-GGTAGTTGCTGTAACGCTGCTTC-3' |
